# Impairment of DNA Methylation Maintenance Is the Main Cause of Global Demethylation in Naive Embryonic Stem Cells

**DOI:** 10.1016/j.molcel.2016.04.025

**Published:** 2016-06-16

**Authors:** Ferdinand von Meyenn, Mario Iurlaro, Ehsan Habibi, Ning Qing Liu, Ali Salehzadeh-Yazdi, Fátima Santos, Edoardo Petrini, Inês Milagre, Miao Yu, Zhenqing Xie, Leonie I. Kroeze, Tatyana B. Nesterova, Joop H. Jansen, Hehuang Xie, Chuan He, Wolf Reik, Hendrik G. Stunnenberg

**Affiliations:** 1 Epigenetics Programme, Babraham Institute, Cambridge CB22 3AT, UK; 2 Department of Molecular Biology, Faculty of Science, Radboud University, 6525GA Nijmegen, the Netherlands; 3 Hematology-Oncology and Stem Cell Transplantation Research Center, Tehran University of Medical Sciences, Tehran, Iran; 4 Department of Chemistry, Department of Biochemistry and Molecular Biology, and Institute for Biophysical Dynamics, The University of Chicago, 929 East 57th Street, Chicago, IL 60637, USA; 5 Howard Hughes Medical Institute, The University of Chicago, 929 East 57th Street, Chicago, IL 60637, USA; 6 Virginia Bioinformatics Institute and Department of Biological Sciences, Virginia Tech, Blacksburg, VA 24060, USA; 7 Department of Laboratory Medicine, Laboratory of Hematology, Radboud University Nijmegen Medical Centre and Radboudumc Institute for Molecular Life Sciences (RIMLS), 6525GA Nijmegen, the Netherlands; 8 Developmental Epigenetics Group, Department of Biochemistry, University of Oxford, South Parks Road, Oxford OX1 3QU, UK; 9 Wellcome Trust Sanger Institute, Hinxton, Cambridge CB10 1SA, UK

## Abstract

Global demethylation is part of a conserved program of epigenetic reprogramming to naive pluripotency. The transition from primed hypermethylated embryonic stem cells (ESCs) to naive hypomethylated ones (serum-to-2i) is a valuable model system for epigenetic reprogramming. We present a mathematical model, which accurately predicts global DNA demethylation kinetics. Experimentally, we show that the main drivers of global demethylation are neither active mechanisms (*Aicda*, *Tdg*, and *Tet1-3*) nor the reduction of de novo methylation. UHRF1 protein, the essential targeting factor for DNMT1, is reduced upon transition to 2i, and so is recruitment of the maintenance methylation machinery to replication foci. Concurrently, there is global loss of H3K9me2, which is needed for chromatin binding of UHRF1. These mechanisms synergistically enforce global DNA hypomethylation in a replication-coupled fashion. Our observations establish the molecular mechanism for global demethylation in naive ESCs, which has key parallels with those operating in primordial germ cells and early embryos.

## Highlights

• Impaired DNA methylation maintenance is the cause of global demethylation in naive ESCs
• Loss of H3K9me2 and UHRF1 lead to impaired maintenance targeting to replication foci
• TET enzymes are not required for global demethylation
• Mathematical model accurately predicts global 5mC and 5hmC during epigenetic resetting

## Graphical Abstract

**Figure:**
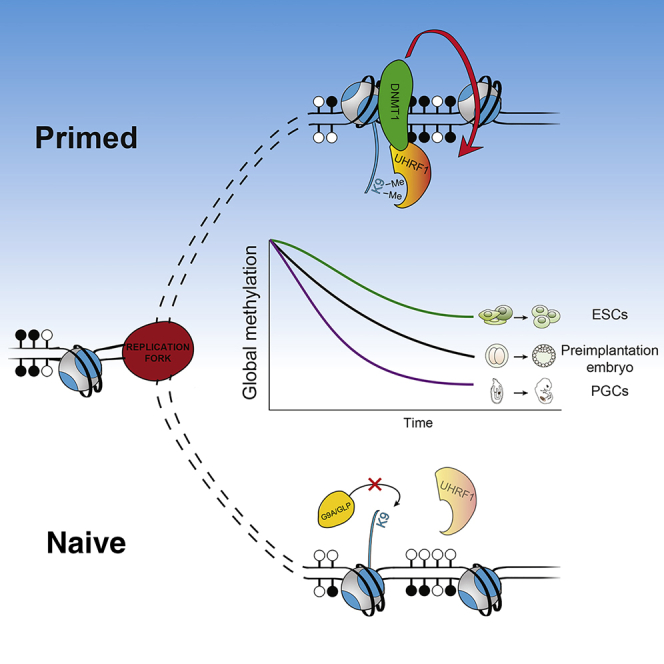

## Teaser

Global demethylation during epigenetic reprogramming in preimplantation embryos, primordial germ cells, and naive embryonic stem cells is a consequence of impaired DNA methylation maintenance and synergistically regulated by a decrease in UHRF1 and H3K9me2 levels, while locus specific methylation maintenance is guided by specific recruitment of UHRF1 to H3K9me2/3. TET enzymes are dispensable for global DNA methylation reprogramming.

## Introduction

Pluripotency describes the transient embryonic potential to form all embryonic germ layers and the germline, excluding the extra-embryonic tissues (Smith, 2001). This state can be recapitulated in vitro in mouse embryonic stem cells (ESCs) derived from the inner cell mass (ICM), maintaining their pluripotent, self-renewing state and the ability to contribute to chimeric embryos (Martello and Smith, 2014). Dictated by the culture conditions, mouse ESCs can adopt two interconvertible states resembling two slightly different in vivo developmental stages. While originally mouse ESCs were grown in serum/leukemia inhibitory factor (LIF)-containing media (“serum ESCs”), recently, serum-free culture conditions have been established that favor derivation and propagation of mouse ESCs in the absence of serum (Ying et al., 2008). These conditions rely on specific inhibition of GSK3beta and Erk1/2 and downstream signaling by two small-molecule inhibitors (“2i ESCs”). 2i ESCs transcriptionally closely resemble cells from the ICM (Nichols and Smith, 2012), and serum ESCs tend to phenocopy cells from the early epiblast and show a greater heterogeneity and differential expression of pluripotency and differentiation factors, resulting in overt and spontaneous differentiation if not held back by LIF (Canham et al., 2010, Singh et al., 2007, Tang et al., 2010). Accordingly, the state of 2i ESCs has been designated the “ground- or naive-state” of pluripotency, and cells grown in 2i are believed to be a much better representation of the cells from the ICM, compared to “primed” serum ESCs (Marks et al., 2012, Martello and Smith, 2014).

In vivo, acquisition of pluripotency in both primordial germ cells (PGCs) and the early embryo coincides with genome-wide epigenetic reprogramming of histone modifications and DNA hypomethylation (Guo et al., 2014, Kobayashi et al., 2012, Kobayashi et al., 2013, Seisenberger et al., 2012, Smith et al., 2014). During the in vitro transition of ESCs from serum to 2i, the epigenome is also globally reprogrammed, with loss of H3K27me3 at repressed promoters, but only minor changes in H3K4me3 and H3K9me3 (Marks et al., 2012). Recently, we and others showed that the genome of 2i ESCs is globally hypomethylated and as such similar to the cells in the ICM, whereas serum ESCs are hypermethylated (Ficz et al., 2013, Habibi et al., 2013, Hackett et al., 2013a, Leitch et al., 2013). To date, the molecular mechanisms regulating this conserved (Guo et al., 2014, Takashima et al., 2014, Wang et al., 2014) genome-wide demethylation in the naive state are unclear.

In the current study, we have revisited this transition and embarked on a comprehensive, time-resolved experimental and mathematical approach to reveal and verify the kinetics and interplay between the different components of the DNA demethylation machinery and to unravel the mechanistic regulation of the main pathway responsible for genome-wide DNA demethylation.

## Results

### Demethylation Dynamics during Serum-to-2i Reprogramming

**Figure 1:**
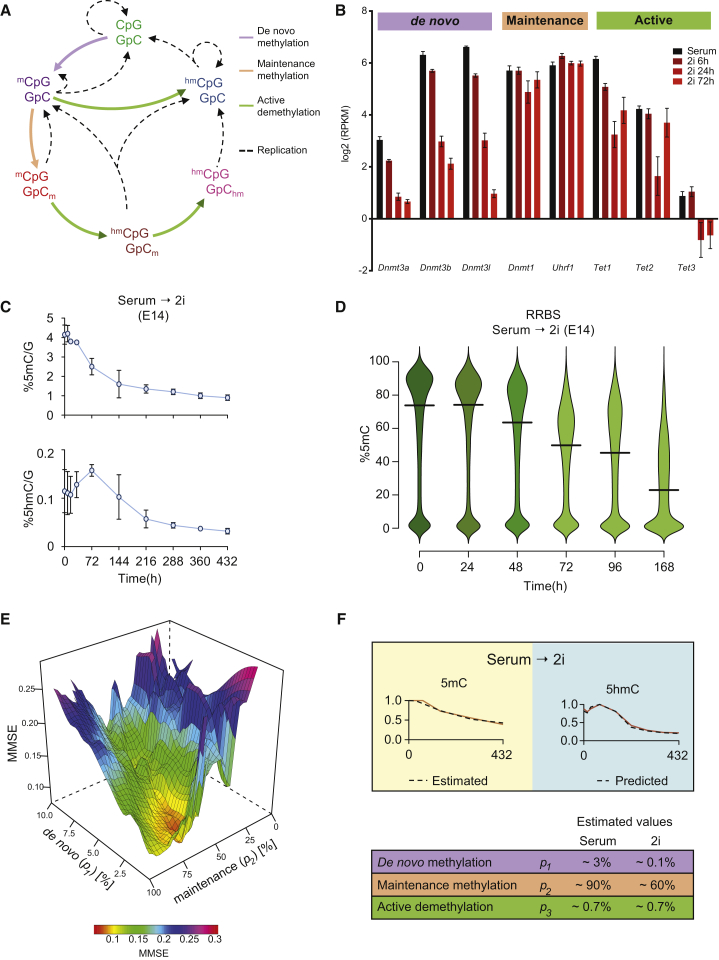
Dynamic Regulation of 5mC and 5hmC during Serum-to-2i Conversion of Mouse ESCs (A) Schematic representation of cytosine methylation/demethylation cycle. Different forms of modified CpG dyads and the corresponding processes are indicated. (B) Expression levels of genes involved in the DNA methylation machinery during serum-to-2i transition. Error bars indicate mean ± SD from three biological replicates. (C) Percentage of 5mC (top) and 5hmC (bottom) as measured by LC-MS in E14 during serum-to-2i conversion. Error bars indicate mean ± SD from three biological replicates. (D) Percentage of 5mC methylation as measured by RRBS in E14 during serum-to-2i conversion. Horizontal bars represent the median values. (E) Graphical representation of the mathematical modeling. The minimum mean square error (MMSE) is plotted against different values for de novo methylation (*p*_*1*_) and maintenance methylation (*p*_*2*_). (F) Overlay of the mathematical model predictions (dotted line) with real measurements (red line) obtained from LC-MS in E14 during serum-to-2i conversion. The table summarizes the results of the mathematical modeling, showing the values estimated for *p*_*1*_, *p*_*2*_, and *p*_*3*_ in the serum steady state and in 2i. *p*_*1*_, proportion of unmethylated CpGs that become hemi-methylated per average cell division; *p*_*2*_, proportion of hemi-methylated CpGs that become fully methylated per average cell division; and *p*_*3*_, proportion of methylated CpGs that become hydroxymethylated per average cell division. See also Figure S1 and Table S1.

DNA demethylation dynamics can be attributed to three major pathways (Wu and Zhang, 2014): (1) maintenance DNA methylation or replication dependent passive dilution, (2) de novo DNA methylation, and (3) active DNA demethylation, primarily via DNA hydroxymethylation (Figure 1A). During the conversion from serum to 2i ESCs, the maintenance methylation components *Dnmt1* and *Uhrf1*, the ten-eleven translocation (TET) enzymes (*Tet1*, *Tet2*, and, at low levels, *Tet3*), *Aicda*, and *Tdg*, which have all been implicated in active DNA demethylation, are expressed at similar transcriptional levels. In contrast, the de novo methylases *Dnmt3a/b* and their regulator *Dnmt3l* are suppressed in the 2i state (Figures 1B and S1A).

To further understand the kinetics of the transition from serum to 2i ESCs, we determined their DNA methylation state at several time points. First, we quantified global levels of 5-methylcytosine (5mC) and 5-hydroxymethylcytosine (5hmC) by liquid chromatography followed by mass spectrometry (LC-MS) (Figure 1C) as well as by reduced representation bisulfite sequencing (RRBS) (Figure 1D), whole-genome bisulfite sequencing (WGBS), and TET-assisted bisulfite sequencing (TAB-seq) (Figures S1B and S1C). In line with our previous studies (Ficz et al., 2013, Habibi et al., 2013), DNA demethylation rapidly ensued after medium replacement (∼32 hr; about two rounds of replication) and thereafter continued gradually, reaching a steady-state level after 14 days (Figure 1C). A moderate increase in 5hmC levels was observed up to 72 hr, suggesting the presence of TET activity.

### Mathematical Modeling of DNA Demethylation Kinetics

To dissect the role and relative contribution of the three pathways and the various regulatory factors involved, we used mathematical modeling to predict DNA demethylation throughout the time course. Since the first population-epigenetic models for DNA methylation dynamics were published (Otto and Walbot, 1990, Pfeifer et al., 1990), several studies were undertaken to improve the predictions by using different approaches and incorporating new biological concepts into the models (Arand et al., 2012, Genereux et al., 2005, McGovern et al., 2012, Sontag et al., 2006). Due to the lack of adequate experimental data describing DNA methylation changes genome-wide, previous descriptive and predictive models could not be fuelled with accurate input values and precise estimates of the parameters. To overcome this obstacle and to obtain accurate input values, we performed genome-wide hairpin bisulfite sequencing (Zhao et al., 2014) and combined these with our other sequencing datasets. We calculated the percentages of fully methylated CpG dyads (mCpG/GpCm), hemi-methylated CpG dyads (mCpG/GpC), and unmethylated CpG dyads (CpG/GpC) (Figure S1D; Table S1) as well as the levels of hydroxymethylated CpGs from TAB-seq data and hairpin bisulfite sequencing. These input values along with the global 5mC values from LC-MS were used to estimate the following parameters, which are directly proportional to the enzyme abundance and/or activity and reflect the amount of substrate that is converted to the product: *p*_*1*_, a dynamic proportionality value for de novo methylation; *p*_*2*_, a proportionality constant for maintenance methylation; and *p*_*3*_, a proportionality constant for active demethylation (hydroxymethylation). Through several iterations of fitting the mathematical model to the 5mC data, we were able to estimate the values of the constants for the serum-to-2i transition in E14 ESCs with the lowest minimum mean square error (MMSE) (Figure 1E). The model also takes into account that the rate of de novo methylation (*p*_*1*_) changes gradually (Figure S1E). Using these values, the model recapitulates 5mC dynamics and predicts the dynamics of 5hmC in ESCs with excellent approximation (R^2^ = 0.99) (Figure 1F, blue panel, dotted line). *p*_*1*_, *p*_*2*_, and *p*_*3*_ reflect the individual activity and overall contribution of the three pathways to the DNA methylation dynamics observed and predicts that maintenance methylation is significantly impaired and a major driver of the DNA demethylation observed (Figure 1F).

### Global Demethylation Kinetics in Mutants of the DNA Methylation Machinery

**Figure 2:**
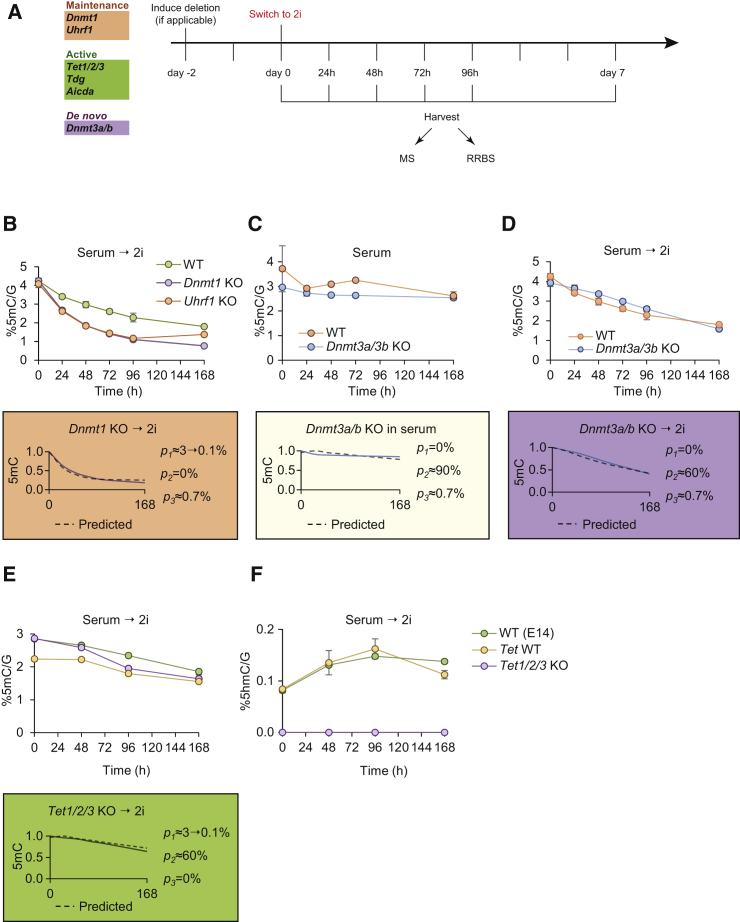
Global Demethylation Kinetics in Mutants of the DNA Methylation Machinery (A) Schematic representation of the experimental setup. Mutants for candidate genes related to global demethylation in the serum-to-2i conversion were initially grown in serum medium. At day 0, culture medium was switched to 2i. Cells were harvested after indicated times. DNA was extracted and analyzed for 5mC and 5hmC levels by LC-MS and/or RRBS. (B) Levels of 5mC in *Uhrf1* and *Dnmt1* KO ESCs during the serum-to-2i transition, measured by mass spectrometry. The colored box shows the overlay between the mathematical model prediction (dotted line) with *p*_*2*_ = 0% and the normalized measured 5mC data. Error bars indicate mean ± SD from three biological replicates. (C) Levels of 5mC in inducible *Dnmt3a/b* KO after induction of deletion in serum media measured by mass spectrometry. The colored box shows the overlay between the mathematical model prediction (dotted line) with *p*_*1*_ = 0% and the normalized measured 5mC data. *p*_*2*_ ≈ 90% since the cells were maintained in serum. Error bars indicate mean ± SD from three biological replicates. (D) Levels of 5mC in inducible *Dnmt3a/b* KO during the serum-to-2i transition. The colored box shows the overlay between the mathematical model prediction (dotted line) with *p*_*1*_ = 0% and the normalized measured 5mC data. Error bars indicate mean ± SD from three biological replicates. (E) Levels of 5mC in *Tet1/2/3* KO and controls during the serum-to-2i transition measured by mass spectrometry. The colored box shows the overlay between the mathematical model prediction (dotted line) with *p*_*3*_ = 0% and the normalized measured 5mC data. Error bars indicate mean ± SD from three biological replicates. (F) Levels of 5hmC in *Tet1/2/3* KO and controls during the serum-to-2i transition measured by mass spectrometry. Error bars indicate mean ± SD from three biological replicates. See also Figure S2.

To validate and fully understand the contribution of the individual DNA methylation and demethylation enzymes in the genome-wide epigenetic reprogramming that characterizes the transition from serum to 2i ESCs, we examined the dynamics of this loss of methylation in mouse embryonic stem cells in which one or more of the components of the DNA methylation machinery had been deleted. To this end, we determined the DNA methylation state at several time points in serum and during the transition from serum to 2i ESCs (Figure 2A) with inducible deletion of *Dnmt1* (*Dnmt1*^fl/fl^); *Uhrf1* (*Uhrf1*^fl/fl^); *Dnmt3a/b* (*Dnmt3a*^fl/fl^ × *Dnmt3b*^fl/fl^) or constitutive deletion of *Aicda* (*Aicda*^−/−^); *Tdg* (*Tdg*^−/−^); *Tet1/2* (*Tet1*^−/−^ × *Tet2*^−/−^); *Tet1/2/3* (*Tet1*^−/−^ × *Tet2*^−/−^ × *Tet3*^−/−^) and in corresponding wild-type control ESCs and compared these to the predictions of our model.

All mutant ESCs tested displayed global loss of 5mC upon serum-to-2i conversion. We specifically compared the rate of demethylation in ESCs lacking either *Dnmt1* or *Uhrf1* with control ESCs and observed an increased rate of demethylation (Figure 2B), showing that loss of DNA methylation maintenance results in increased demethylation rates. This supports the prediction from the mathematical model (dotted lines in the colored boxes) and implicates a failure of DNA methylation maintenance in 2i ESCs, albeit not a complete loss. Next, we compared the demethylation kinetics in ESCs lacking *Dnmt3a* and *Dnmt3b*. Interestingly, deletion of *Dnmt3a/b* in serum grown ESCs results in only a marginal decrease in the genomic level of 5mC (Figure 2C), and the kinetics of DNA demethylation are unaltered in the serum-to-2i conversion (Figure 2D), showing that loss of de novo methylation is not responsible for global loss of DNA methylation. Finally, we assessed the contribution of enzymes involved in active demethylation pathways in the serum-to-2i conversion. As predicted by the model, ESCs lacking *Tet1/2/3*, *Tdg*, or *Aicda* showed strikingly similar demethylation dynamics to their wild-type control counterparts (Figures 2E, S2A, and S2B). Since TET-driven oxidation has been previously suggested as a potential driver of demethylation in the serum-to-2i conversion, we confirmed the loss of 5hmC in *Tet1/2/3* knockout (KO) cells (Figure 2F). This shows that the TET enzymes are actively oxidizing 5mC during the serum-to-2i conversion but are neither sufficient nor necessary for global DNA demethylation.

### Locus-Specific Involvement of TET-Dependent Demethylation

**Figure 3:**
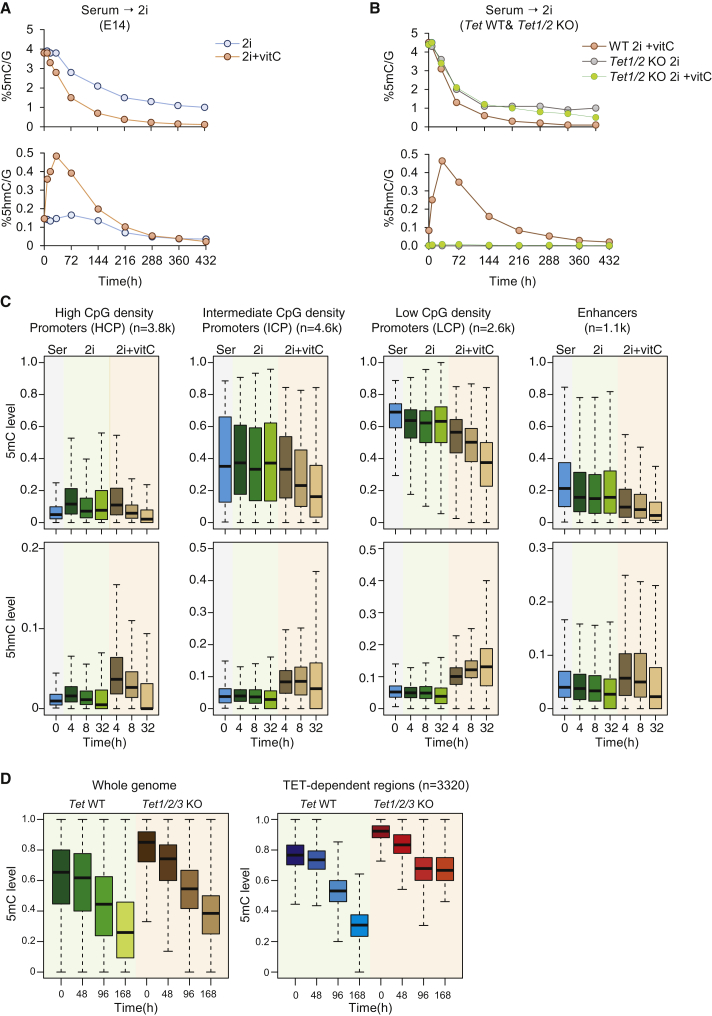
TET-Dependent Demethylation Dynamics during Reprogramming of Serum-to-2i ESCs (A) Percentage of 5mC (top) and 5hmC (bottom) as measured by LC-MS in E14 during serum-to-2i transition in the absence (blue line) or presence of vitC (red line). Error bars indicate mean ± SD from three biological replicates. (B) Percentage of 5mC (top) and 5hmC (bottom) as measured by LC-MS in *Tet1/2* KO during serum-to-2i transition in the absence (gray line) or presence of vitC (green line). Error bars indicate mean ± SD from three biological replicates. (C) Distribution of 5mC (top) and 5hmC levels (bottom) during the first 32 hr of reprogramming from serum (gray) to 2i (green) or 2i+vitC (orange) measured by WGBS and TAB-seq over high-CpG promoters (HCP), intermediate-CpG promoters (ICP), low-CpG promoters (LCP) and enhancers. For each class of genomic element, only the subset having 5hmC enrichment in serum (T = 0 hr) was considered. The horizontal line within the box plots represents the median. (D) Average methylation levels over 500-bp tiles in *Tet1/2/3* KO cells and corresponding control cells (*Tet* WT). Tet-dependent regions were identified using k-means clustering. See also Figure S3 and Table S2.

The increased levels of 5hmC during the serum-to-2i conversion as well as publications showing further TET-dependent hypomethylation in vitamin C (vitC)-treated 2i ESCs (Blaschke et al., 2013, Chung et al., 2010, Yin et al., 2013) raised the possibility that TET proteins could contribute to the observed demethylation dynamics. We determined the 5mC and 5hmC levels of ESCs at several time points during the serum-to-2i conversion in the presence or absence of vitC (Figure 3A) and observed a significant increase in 5hmC upon vitC treatment, which resulted in an accelerated rate of demethylation and further global hypomethylation. To confirm that this effect was TET dependent, we measured the levels of 5mC and 5hmC in *Tet1/2* KO cells in the presence or absence of vitC (Figure 3B), showing that the increased rate of demethylation (and hydroxymethylation) is dependent on the activity of TET proteins. Using the values of *p*_*1*_ and *p*_*2*_ from E14 ESCs (Figure 1F), we estimated an ∼4-fold increase of active demethylation (*p*_*3*_) in the presence of vitC. Mathematical modeling of the dynamics of 5hmC accurately predicts the observed 5hmC levels during the serum-to-2i conversion in the presence of vitC (Figure S3A).

Having established that exclusively in the presence of vitC TET proteins contribute to global loss of 5mC in the serum-to-2i conversion, we asked whether TET-dependent hydroxymethylation is essential for locus specific demethylation. We performed TAB-seq in parallel to WGBS on the same DNA during the early phase of the time course in the presence or absence of vitC (Figure S3B) as well as RRBS from *Tet1/2/3* KO cells and corresponding wild-type control ESCs during different time points of the serum-to-2i conversion (Figure S3C). These datasets confirmed that TET-dependent hydroxymethylation does not significantly contribute to global demethylation, unless their activity was enhanced by vitC (∼3-fold increase in 5hmC in the first 32 hr). We analyzed the distribution of 5mC and 5hmC over functionally distinct genomic regions with 5hmC enrichment in serum (Figure 3C) and stratified promoters into high-, intermediate-, and low-CpG-density promoters (HCP, ICP, and LCP, respectively) (Weber et al., 2007). The majority of HCPs (n = 3,121) are very low methylated in serum, and only a small subgroup are intermediate (n = 143) or intermediate-low (n = 563) methylated, containing some germ cell-specific genes (e.g., *Dazl*, *Prdm14*, and *Dppa3*) (Figure S3D). In the absence of vitC, we observe only minor if any change in 5mC/5hmC levels over any of the three classes of promoters. In the presence of vitC, however, conversion of 5mC to 5hmC is already apparent as early as 4 hr, and the speed of 5mC loss correlates with CpG density and is different for each class. The kinetics of 5mC to 5hmC conversion and subsequent loss of 5hmC (73% loss) is fastest at HCPs, while ICPs show less dramatic changes (32% loss), with substantial loss of 5hmC being apparent only later. At LCPs, 5hmC slowly accumulates over the time period investigated. Enhancers, defined as elements overlapping H3K4me1, H3K27ac, and DNaseI hypersensitivity and excluding transcription start sites (TSS) (±2 kb) in E14 serum ESCs, show 10%–40% 5mC levels in serum ESCs, in line with previous studies showing that enhancers have low abundance of 5mC (Stadler et al., 2011) and follow kinetics similar to ICPs. The largest part of the genome behaves similar to LCPs; conversion to 5hmC and subsequent erasure is very slow (Figure 3C).

Finally, we analyzed the methylome in *Tet1/2/3* KO cells. Using k-means clustering on average DNA methylation over 500-bp tiles across the genome, we found a cluster of tiles (n = 3,770) that maintains methylation in *Tet1/2/3* KO cells, but not in the corresponding wild-type control cells (Figure 3D). These regions have higher 5mC levels in the *Tet1/2/3* KO cells, but we could not identify any significant functional enrichment associated with them (Table S2).

### UHRF1 Is Downregulated at the Protein Level during the Serum-to-2i Transition

The mathematical model had predicted that maintenance methylation is significantly impaired and a major driver of the DNA demethylation observed (Figure 1F). Subsequently, we confirmed the rapid demethylation upon deletion of *Dnmt1* or *Uhrf1* (Figure 2B) and also showed that loss of de novo methylation does not explain the demethylation dynamics observed (Figures 2C and 2D). In order to understand the mechanistic regulation of this impairment, we focused on the individual components of the maintenance methylation machinery, in particular on the role of *Uhrf1*.

**Figure 4:**
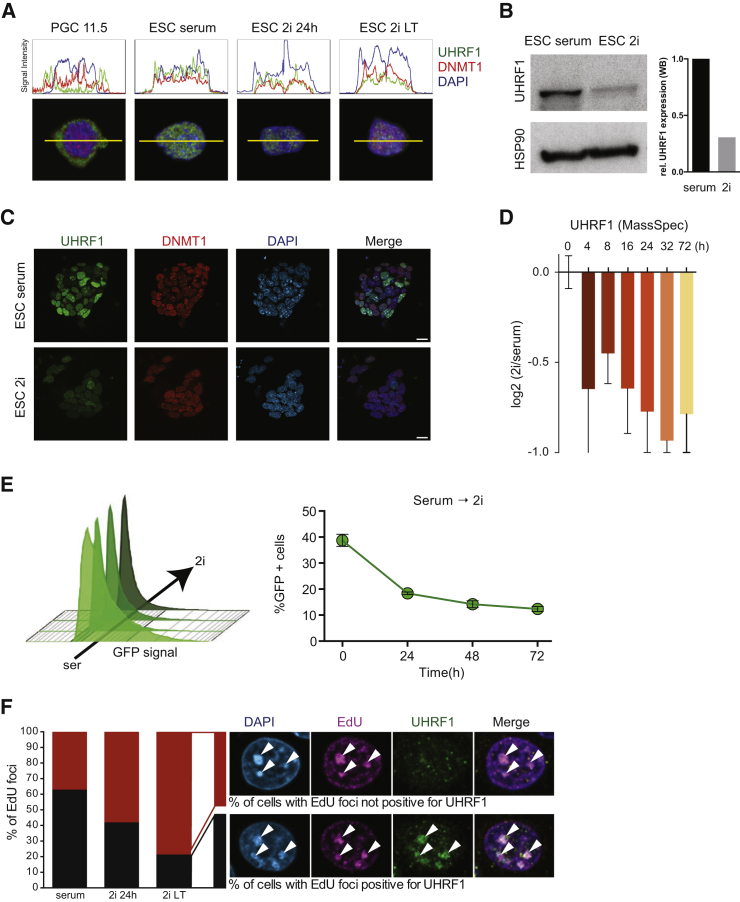
UHRF1 Is Regulated at the Protein Level in 2i (A) Cellular localization of UHRF1 (green) and DNMT1 (red) in mouse PGCs (E11.5), serum ESCs, 24-hr 2i ESCs, and long-term (LT) 2i ESCs. DAPI (blue) labels the nucleus. The RGB profiles show the signal intensity for each pixel along the horizontal yellow line. (B) Western blot analysis for UHRF1 protein in serum and LT 2i ESCs. Relative quantifications of the signal intensities of the bands on the western blot are shown in the bar chart. (C) Immunofluorescence staining for UHRF1 (green), DNMT1 (red) and DAPI (blue) in serum and LT 2i ESCs. Scale bar represents 20 μm. (D) Protein levels of UHRF1 detected by protein mass spectrometry (label-free quantification [LFQ]) at different time points during serum-to-2i conversion, relative to serum ESCs. Error bars indicate mean ± SD from two biological replicates. (E) FACS analysis of UHRF1-GFP fusion protein in ESCs during serum-to-2i conversion. Histograms show the GFP signal intensity at different time points during the conversion. The black arrow depicts the threshold used to quantify the percentage of GFP+ cells, shown in the line graph. Error bars indicate mean ± SD from three biological replicates. (F) Percentage of replication foci with co-localization of UHRF1 in serum, 24-hr 2i and LT 2i ESCs. Images from serum ESCs showing examples for the two quantified states; arrows indicate EdU+ replication foci. See also Figure S4.

In primordial germ cells, maintenance methylation was reported to be impaired in part by nuclear exclusion of UHRF1 (Seisenberger et al., 2012). We analyzed the subcellular localization of UHRF1 and DNMT1 in ESCs grown in serum and 2i (Figure 4A) and did not detect any nuclear exclusion of either UHRF1 or DNMT1 in ESCs. However, we observed a reduction in the signal intensity of UHRF1 protein in the 2i samples. Our initial transcriptomic analysis of the cells undergoing serum-to-2i transition (Figure 1B) did show that the expression of *Dnmt3a* and *Dnmt3b*, together with the catalytically inactive regulatory isoform *Dnmt3l*, was substantially reduced in 2i, while *Uhrf1* mRNA levels were unchanged. In contrast, UHRF1 protein levels were significantly reduced (∼3-fold by western blot; 2-fold by quantitative mass spectrometry) in 2i ESCs (Figures 4B–4D), as were the levels of the de novo DNMTs (Figure S4A). We found heterogeneous expression of UHRF1 in serum and 2i ESCs (Figure 4C), which did not correlate with the expression of the pluripotency marker NANOG (Figure S4B) but can be attributed to the cell-cycle-dependent regulation of UHRF1 (Bonapace et al., 2002). To further confirm the observation that UHRF1 is regulated at the protein level, we generated an ESC line with constitutive overexpression of an UHRF1-GFP fusion protein. Similar to our observations on endogenous UHRF1, fluorescence-activated cell sorting (FACS) analysis of the UHRF1-GFP cell line showed that UHRF1-GFP was expressed in serum ESCs but was rapidly lost upon serum-to-2i conversion (Figures 4E and S4C), while mRNA levels of endogenous *Uhrf1* and exogenous *Uhrf1-Gfp* remained stable (Figure S4D). Interestingly, we also observed a rapid increase of UHRF1-GFP protein levels upon transfer back to serum growth conditions (Figure S4E), suggesting that the changed signaling environment in 2i specifically affects UHRF1 protein stability.

To confirm whether the regulation of UHRF1 at the protein level was also relevant in human ESCs, we assessed mRNA and protein levels in conventional and naive (Takashima et al., 2014) human ESCs by immunofluorescence (IF). Similar to our observations in mouse ESCs, *UHRF1* and *DNMT1* mRNA levels are not changed between the two states (Figure S4F), but UHRF1 protein levels were significantly reduced (∼2-fold reduced) in the naive cells (Figure S4G), pointing to a conserved regulation of UHRF1 in naive ESCs.

### UHRF1 Recruitment to Replication Foci Is Impaired in 2i ESCs

The rapid, though not complete, loss of UHRF1 protein in 2i conditions prompted us to ask whether recruitment of the DNA maintenance methylation machinery to the replication forks was impaired. Previous IF experiments in cells lacking Uhrf1 proved that the presence of UHRF1 is absolutely required for recruitment of DNMT1 to replication foci (Sharif et al., 2007). We labeled replication foci by incubating serum or 2i ESCs for 8 min prior to fixation with 5-ethynyl-2′-deoxyuridine (EdU), which is incorporated into newly synthesized DNA. Co-staining for UHRF1 and EdU allowed us to count the number of cells with EdU-positive foci for co-localization of UHRF1 with replication foci. While UHRF1 co-localizes with EdU-positive replication foci in the majority of cells in serum conditions, this number is significantly reduced under 2i conditions (Figure 4F). The recruitment of UHRF1 to EdU-positive replication foci is further reduced with time in 2i. These results suggest that DNA demethylation in the serum-to-2i conversion is a consequence of impaired DNA methylation maintenance through impaired recruitment of DNMT1 by UHRF1 to the replication fork.

### H3K9me2 Is Reduced in 2i ESCs

**Figure 5:**
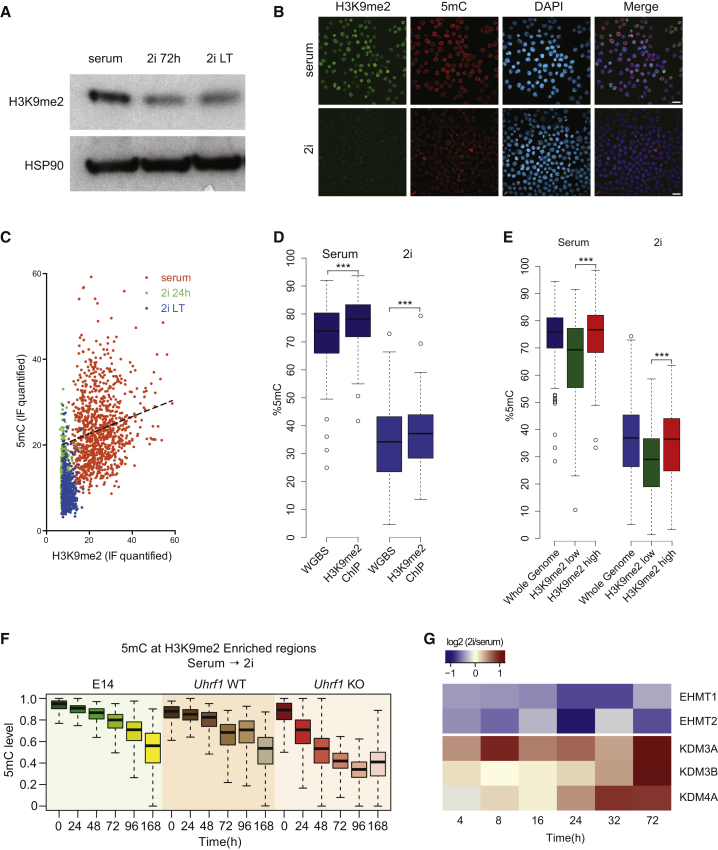
H3K9me2 Is Rapidly Reduced in 2i ESCs (A) Western blot analysis for H3K9me2 in serum and 2i ESCs. (B) Immunofluorescence staining for H3K9me2 (green), 5mC (red) and DAPI (blue) in serum and LT 2i ESCs. Scale bar represents 20 μm. (C) Correlation of the quantified signals of H3K9me2 and 5mC in immunofluorescence staining of serum, 24-hr 2i, and LT 2i ESCs. The dashed line represents the linear regression between H3K9me2 and 5mC signals in serum ESCs. (D) 5mC levels in H3K9me2-bound DNA and corresponding input samples from serum and LT 2i ESCs, measured by H3K9me2-ChIP-BS-seq. (E) 5mC levels in regions with high or low H3K9me enrichment in serum and LT 2i ESCs. (F) Levels of 5mC, determined by RRBS at H3K9me2 enriched regions during the serum-2i transition in E14, *Uhrf1* WT, and *Uhrf1* KO ESCs. (G) Relative protein levels of known H3K9 modifiers detected by protein mass spectrometry (label-free quantification [LFQ]) at different time points during serum-to-2i conversion. Levels are normalized to serum ESCs. Student’s t test was performed for the indicated comparisons (^∗∗∗^p < 0.001). See also Figure S5.

The drastic impairment of recruitment of UHRF1 to replication sites was surprising, as the levels of UHRF1 were reduced albeit still detectable in 2i conditions. The recruitment of UHRF1 depends on the recognition of both hemimethylated CpGs via its SRA domain and of methylated lysine 9 on histone 3 (H3K9) through its Tudor domain (Citterio et al., 2004, Karagianni et al., 2008, Rothbart et al., 2012). Loss of H3K9 methyltransferase G9a results in loss of global DNA methylation (Zhang et al., 2016), and similarly, it has been concluded that loss of H3K9 methylation, in particular H3K9me2, is an additional key step leading to genome-wide demethylation in PGCs (Kurimoto et al., 2015). H3K9me3 localization is relatively unchanged between serum and 2i ESCs and mainly enriched at repetitive elements (Marks et al., 2012), but the importance of loss of H3K9me2 is not clear (Walter et al., 2016). We found a significant reduction in the levels of H3K9me2 upon serum-to-2i conversion (∼2-fold reduction) (Figures 5A, 5B, and S5A). Interestingly, loss of H3K9me2 was rapid, and we detected a weak correlation by IF between the global levels of H3K9me2 and DNA methylation in serum ESCs (Figure 5C), in line with the notion that H3K9me2-dependent recruitment of UHRF1 plays a key role in DNA methylation maintenance in ESCs, possibly explaining the drastic impairment of recruitment of UHRF1 to the replication foci.

To understand any locus-specific effects of the loss of H3K9me2, we performed H3K9me2 chromatin immunoprecipitation sequencing (ChIP-seq) analysis. While the overall pattern of H3K9me2 enrichment did not change during the serum-to-2i conversion, regions with low H3K9me2 enrichment further flattened out (Figure S5B). To correlate the presence of H3K9me2 with the levels of 5mC, we performed H3K9me2 chromatin immunoprecipitation followed by bisulfite sequencing (ChIP-BS-seq) (Brinkman et al., 2012). The 5mC levels in serum ESCs as well as 2i ESCs were significantly increased in the H3K9me2-bound DNA when compared to corresponding input samples (Figure 5D). We then divided the genome into regions with high or low H3K9me2 enrichment and found a significant increase in the 5mC levels at H3K9me2-high regions (Figure 5E).

Next, we generated RRBS libraries of *Uhrf1*^−/−^ and corresponding wild-type control ESCs during the serum-to-2i conversion (Figure S5C) to identify regions that are only maintained in the presence of UHRF1. Computational analyses of these and E14, *Tet1/2/3* KO, and wild-type control lines identified a number of genomic regions resistant to DNA demethylation in wild-type cells, but not upon deletion of *Uhrf1* (Figure S5D; blue cluster), indicating strong locus specific recruitment. These regions are enriched for H3K9me2 in 2i ESCs and for H3K9me3 in 2i and serum ESCs (Figures S5E and 5F). We next measured 5mC levels over H3K9me2- (Figure 5F) and H3K9me3-enriched (Figure S5G) regions (Marks et al., 2012) and found that these regions retain high levels of DNA methylation in 2i ESCs in an UHRF1-dependent manner.

Finally, we asked how H3K9me2 levels were regulated during the serum-to-2i conversion. We quantified mRNA levels of key H3K9 methylases and demethylases by RNA-seq (Figure S5H) and protein levels by mass spectrometry (Figure 5G). Interestingly, we found that several H3K9 demethylases, including KDM3A, KDM3B, and KDM4A, were upregulated specifically at the protein level in 2i conditions. Conversely, a number of H3K9 methylases, such as EHMT1 (GLP) and EHMT2 (G9A), were downregulated.

## Discussion

**Figure 6:**
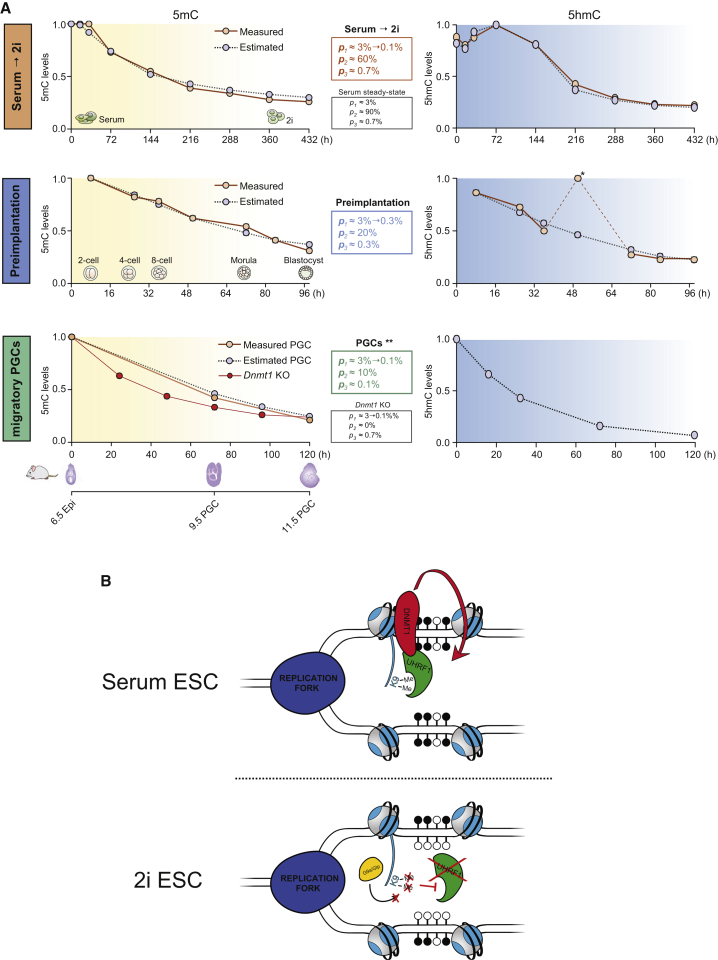
Dual Regulation of DNA Demethylation during Serum-2i Transition (A) Mathematical modeling of serum-2i transition, preimplantation development, and migratory PGC demethylation. The model estimates the activity of the main pathways related to DNA methylation dynamics, i.e., de novo methylation (*p*_*1*_), maintenance methylation (*p*_*2*_), and active demethylation (*p*_*3*_). The first column shows measured (solid lines) and estimated (dotted lines) global levels 5mC (values have been scaled) during serum-2i transition, preimplantation development, and migratory PGC demethylation. The second column summarizes the estimated activities of the three pathways in each condition. The steady-state activities in serum ESCs are included as a comparison. The third column shows the predicted (dotted lines) global 5hmC values (scaled) and, if available, also measured (solid lines) 5hmC levels. ^∗^The mathematical modeling did not predict a peak of global 5hmC levels in eight-cell embryos (Okamoto et al., 2016). ^∗∗^Global 5mC dynamics in migratory PGCs can be can be explained by two solutions of the mathematical model. The first solution is shown here and the second solution is shown in Figure S6. (B) Cartoon representation of the proposed model. In serum cells, UHRF1 is recruited to replication foci by binding to hemi-methylated DNA and H3K9me2 chromatin marks. Switching to 2i conditions results in passive loss of 5mC driven by a multilayer of regulation: downregulation of UHRF1 at the protein level, and loss of H3K9me2, which results in impaired recruitment to replication foci. See also Figure S6.

Genome-wide DNA methylation erasure is a distinguishing epigenetic feature of preimplantation embryos (Guo et al., 2014, Smith et al., 2014, Wang et al., 2014), developing PGCs (Gkountela et al., 2015, Guo et al., 2015, Hackett et al., 2013b, Seisenberger et al., 2012, Tang et al., 2015), and naive ESCs (Ficz et al., 2013, Habibi et al., 2013, Leitch et al., 2013, Takashima et al., 2014). Here, we have developed a mathematical model that is able to accurately estimate 5mC levels and the individual activity of the three main pathways relevant to DNA methylation dynamics (*p*_*1*_, de novo; *p*_*2*_, maintenance; and *p*_*3*_, active demethylation) and predicts with great accuracy corresponding 5hmC kinetics in all instances of global epigenetic reprogramming. Validation of the model in different mutant ESC lines during serum-to-2i conversion demonstrates the power and the high prediction accuracy of the algorithm. Using methylation levels (5mC) from previously published datasets for migratory PGCs (E6.5–E11.5) (Seisenberger et al., 2012) and preimplantation embryos (two-cell stage to E4) (Okamoto et al., 2016, Wang et al., 2014), we have estimated the in vivo activity of the DNA methylation pathways and the dynamics of 5hmC levels during these phases of global DNA demethylation (Figures 6A and S6). All examples of global demethylation in the mouse can hence be accurately modeled and the predicted activities of the three pathways are mirrored by the levels and regulation of the enzymes and factors involved (where this is known). This leads to a unified view of genome-wide demethylation mechanisms.

In all instances of epigenetic reprogramming, global methylation is lost in a replication-dependent manner as a consequence of impaired maintenance methylation (*p*_*2*_). The individual maintenance activity dictates the rate of global demethylation, which is fastest in PGCs, where the rate of demethylation is almost as fast as that brought about by complete loss of maintenance methylation (*Dnmt1* KO ESCs). This result agrees with the observations made in preimplantation embryos, where an oocyte-specific cytoplasmic DNMT1 isoform is predominantly expressed and the somatic DNMT1 isoform is only expressed at low levels, but shows some activity, in particular at imprinted regions (Cirio et al., 2008, Dean, 2008, Hirasawa et al., 2008). Similar to 2i ESCs, the model predicts a significant impairment of de novo methylation in preimplantation embryos and migratory PGCs (*p*_*1*_, ∼10- and 30-fold reduction in activity) when compared to the steady-state levels found in serum ESCs. This is in agreement with substantial downregulation of DNMT3A/B in PGCs (Hackett et al., 2013b, Seisenberger et al., 2012, Wu and Zhang, 2014) and naive ESCs (Ficz et al., 2013, Habibi et al., 2013) and suggests that de novo methylation is not active prior to E4 in early embryos and the expression of de novo DNMTs is only needed for remethylation in the epiblast (Guenatri et al., 2013, Hirasawa et al., 2008). Finally, the importance of active demethylation (*p*_*3*_) for global loss of DNA methylation is estimated to be very low in all instances of epigenetic reprogramming. The 5hmC predictions of the model recapitulate 5hmC dynamics in ESCs and in preimplantation embryos (with the exception of a reported peak at the eight-cell stage) and also predict 5hmC dynamics in PGCs (Figures 6A and S6). Noteworthy, the dynamics in migratory PGCs can be explained by two solutions, which both accurately mimic 5mC levels but predict different 5hmC dynamics. One solution predicts that both maintenance methylation and TET activity are strongly reduced (“impaired maintenance methylation”) (Figure 6A), while the second solution predicts that maintenance methylation is only ∼2-fold reduced and TET activity is high (“TET-dependent demethylation”) (Figure S6). In mouse PGCs, UHRF1 levels are significantly decreased and high levels of hemi-methylated CpG dyads were found (Seisenberger et al., 2012), both supporting a strong impairment of maintenance methylation. In addition, loss of H3K9me2 was reported in in vivo and in vitro PGCs (Kurimoto et al., 2015, Seki et al., 2005) and knockdown of *Tet1* and *2* did not affect global DNA demethylation in vitro (Vincent et al., 2013). Thus, we reason that the “impaired maintenance methylation” model (Figure 6A) faithfully recapitulates the in vivo situation.

Mechanistically, we have dissected the different pathways relevant to DNA methylation dynamics in primed (serum) and naive (2i) ESCs (Wu and Zhang, 2014) and shown that global demethylation is, contrary to previous assumptions, a consequence of neither loss of de novo methylation nor active Tet-dependent demethylation but caused by impaired maintenance methylation. DNMT1 is recruited to the replication fork through UHRF1, which itself interacts with both hemimethylated CpG dyads and methylated H3K9 and loss of either interaction impairs recruitment (Liu et al., 2013). In naive mouse and human ESCs, protein levels of UHRF1 are reduced, limiting the recruitment of DNMT1. In addition, loss of H3K9me2 further reduces UHRF1 recruitment and in turn impairs DNA methylation maintenance by DNMT1 (Figure 6B). UHRF1 is regulated at the protein level in human and mouse ESCs, as well as in human and mouse PGCs (Gkountela et al., 2015, Pastor et al., 2016, Seisenberger et al., 2012, Tang et al., 2015). Interestingly, in mouse PGCs there is also transcriptional silencing of *Uhrf1* (Kurimoto et al., 2015, Magnúsdóttir et al., 2013, Nakaki et al., 2013, Seisenberger et al., 2012), which may well underlie the considerably faster demethylation kinetics in mouse PGCs when compared to naive ESCs. Hence, several pathways have apparently evolved that modulate maintenance methylation by controlling UHRF1 activity at different levels. The additional level of transcriptional regulation in mouse PGCs suggests that the short time frame of epigenetic resetting in the mouse germline necessitated an additional regulatory layer, ensuring almost complete impairment of maintenance methylation and thus rapid demethylation (Figure 6A). This is in contrast to human PGCs, where demethylation occurs with slower, potentially intra-individual-specific kinetics (von Meyenn and Reik, 2015).

In addition to the reduction of UHRF1 protein levels, the signaling changes imposed by 2i in ESCs (MAPK and GSK3 signaling inhibition) elicit a decrease in the levels of H3K9me2, thereby synergistically driving genome-wide DNA demethylation, while allowing methylation maintenance of specific genomic loci. This regulation of H3K9me2 is also evident in PGCs, with apparent loss of H3K9me2 in mouse (Kurimoto et al., 2015, Seki et al., 2005) and human PGCs (Tang et al., 2015). We found that regions with high levels of H3K9me2 in naive ESCs are also characterized by higher level of DNA methylation (Figure 5F) and regions resistant to DNA demethylation in the presence of UHRF1 show significant enrichment of H3K9me2 (Figure S5E). We propose that in the face of global demethylation, locus-specific enrichment of H3K9me2/3 allows DNA methylation maintenance at specific regions via recruitment of UHRF1. Similarly, while TET enzymes were dispensable for global epigenetic reprogramming, TET-dependent demethylation was evident at a limited number of specific loci. WGBS and TAB-seq experiments revealed that active demethylation is restricted to a small proportion of the genome, predominantly including active promoters and enhancers.

Taken together, loss of DNA methylation in ESCs, PGCs, and preimplantation embryos is the result of the concerted regulation of two pathways, namely reduction of UHRF1 protein levels and global loss of H3K9me2, thereby impairing recruitment of DNMT1 to the replication fork. This elegant mechanism facilitates the maintenance of methylation at specific loci while the genome is simultaneously demethylated on a global scale.

## Experimental Procedures

### Cell Culture

Mouse ESCs were cultured in the presence of LIF in DMEM containing 15% fetal calf serum. A tamoxifen-inducible Cre recombinase was used to induce recombination of *loxP* sites in the floxed cell lines 48 hr prior to the experiment. For serum-to-2i transition, serum medium was replaced by serum-free N2B27 supplemented with LIF, mitogen-activated protein kinase kinase (MEK) inhibitor PD0325901 (1 μM), and GSK3 inhibitor CHIR99021 (3 μM).

### Mass Spectrometry of Nucleosides

Mass spectrometry of nucleosides was performed as previously described (Ficz et al., 2013, Kroeze et al., 2014). Briefly, 150–1,000 ng genomic DNA was digested using DNA Degradase Plus (Zymo Research) according to the manufacturer’s instructions and analyzed by liquid chromatography-tandem mass spectrometry.

### Next-Generation Sequencing

WGBS, RRBS, and TAB-seq were performed as described previously (Lister et al., 2008, Smallwood et al., 2011, Yu et al., 2012). Whole-genome hairpin bisulfite sequencing was performed as described previously (Zhao et al., 2014). Chromatin immunoprecipitation of serum and 2i ESCs was performed using anti-H3K9me2 antibodies (Abcam, ab1220). Eluted DNA was then used for ChIP-seq library preparation using the MicroPlex Library Preparation Kit v2 (Diagenode) or alternatively treated as WGBS samples as described above.

See also Supplemental Experimental Procedures.

## Author Contributions

F.v.M., M.I., and E.H. performed the experiments, analyzed the data, and carried out bioinformatics analysis. F.S. performed immunofluorescence experiments and analysis. E.P. carried out bioinformatics analysis. N.Q.L. performed proteomic analysis. E.H. and A.S.Y. performed mathematical modeling. M.Y. and C.H. prepared WGBS and TAB-seq libraries. Z.X. and H.X. performed hairpin-bisulfite library preparation. I.M. generated *Aicda*^−/−^ cells, and T.B.N. generated *Dnmt[3a*^*fl/fl*^*,3b*^*fl/fl*^*]* cells. L.I.K. and J.H.J. performed 5mC/5hmC LC-MS analysis. F.v.M., M.I., E.H., H.G.S., and W.R. designed the experiments and wrote the manuscript. H.G.S. and W.R. provided supervision.

## Accession Numbers

The accession numbers for the next-generation-sequencing data reported in this study are GEO: GSE70724 and GEO: GSE77420.

## Supplemental Information

Document S1. Supplemental Experimental Procedures, Figures S1–S6, and Table S1

Table S2. List of 500-bp Regions Higher Methylated in TET1/2/3 KO Cells Compared to TET Wild-type Cells, Related to Figures 3 and S3 Reads were mapped to the mouse NCBIM37 genome build using Bismark and average CpG methylations were calculated over each 500-bp region in Seqmonk.

Document S2. Article plus Supplemental Information
