# Impairment of DNA Methylation Maintenance Is the Main Cause of Global Demethylation in Naive Embryonic Stem Cells

**DOI:** 10.1016/j.molcel.2016.06.005

**Published:** 2016-06-16

**Authors:** Ferdinand von Meyenn, Mario Iurlaro, Ehsan Habibi, Ning Qing Liu, Ali Salehzadeh-Yazdi, Fátima Santos, Edoardo Petrini, Inês Milagre, Miao Yu, Zhenqing Xie, Leonie I. Kroeze, Tatyana B. Nesterova, Joop H. Jansen, Hehuang Xie, Chuan He, Wolf Reik, Hendrik G. Stunnenberg

(Molecular Cell *62*, 848–861; June 16, 2016)

In the original version of the above article, a recent publication and its findings had not been acknowledged. The online and print versions have now been corrected, and the corrected sentence reads “… but the importance of loss of H3K9me2 is not clear (Walter et al., 2016).” The full citation to the reference has been added to the reference list: Walter, M., Teissandier, A., Pérez-Palacios, R., Bourc’his, D. (2016). An epigenetic switch ensures transposon repression upon dynamic loss of DNA methylation in embryonic stem cells. Elife 5. http://dx.doi.org/10.7554/eLife.11418

