# Supplementary material for: Impairment of DNA Methylation Maintenance Is the Main Cause of Global Demethylation in Naive Embryonic Stem Cells
Source: Mol Cell. 2016 Jun 16;62(6):848–61. doi: 10.1016/j.molcel.2016.04.025 (PMC4914828; doi:10.1016/j.molcel.2016.04.025)
Supplement: Document S1. Supplemental Experimental Procedures, Figures S1–S6, and Table S1 [file mmc1.pdf]

**Molecular Cell, Volume *62***

## Supplemental Information

# Impairment of DNA Methylation Maintenance Is the Main Cause of Global Demethylation in Naive Embryonic Stem Cells

**Ferdinand von Meyenn, Mario Iurlaro, Ehsan Habibi, Ning Qing Liu, Ali Salehzadeh-Yazdi, Fátima Santos, Edoardo Petrini, Inês Milagre, Miao Yu, Zhenqing Xie, Leonie I. Kroeze, Tatyana B. Nesterova, Joop H. Jansen, Hehuang Xie, Chuan He, Wolf Reik, and Hendrik G. Stunnenberg**

Figure S1

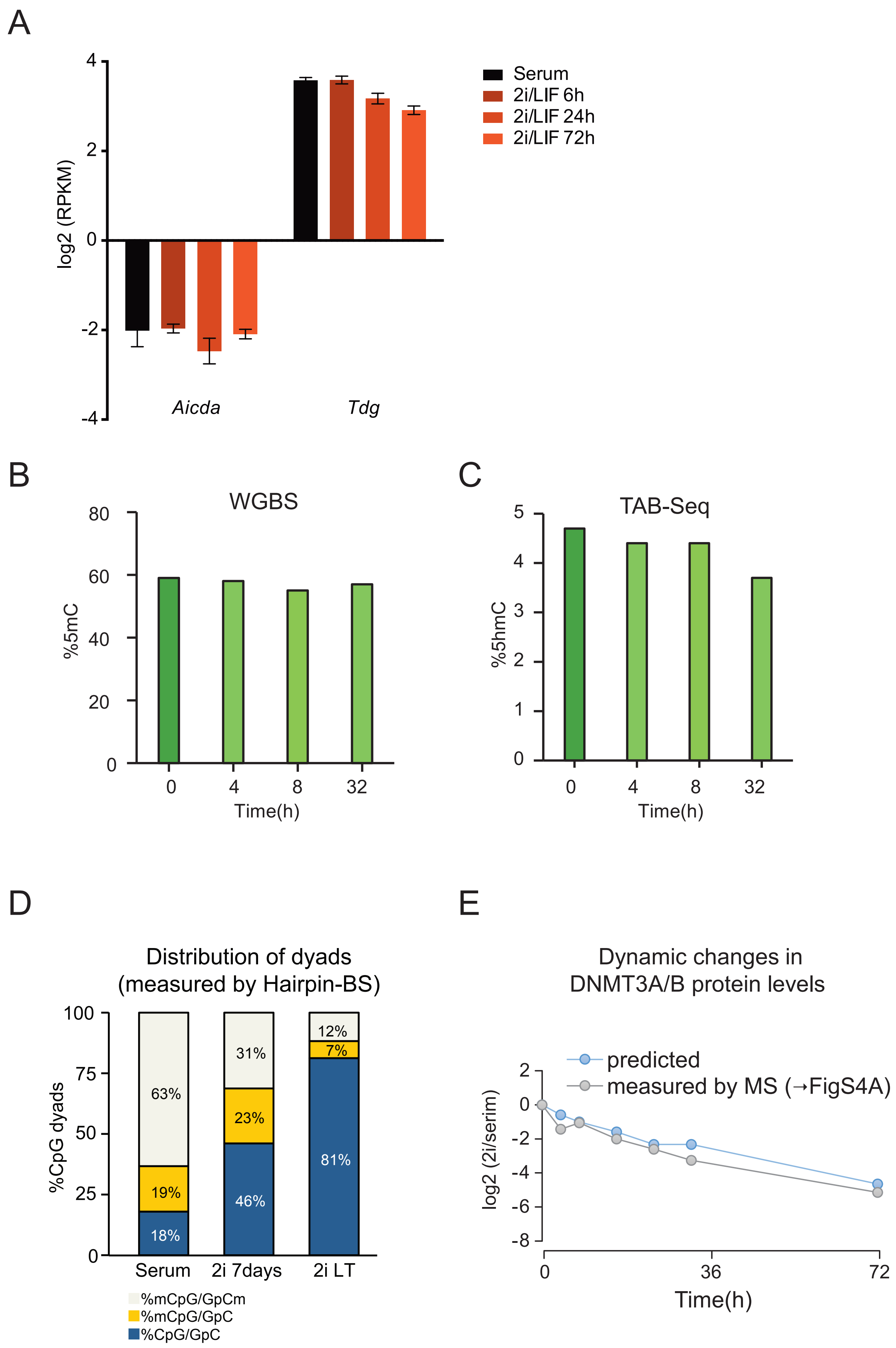
A
4
2
0
-2
-4
log2 (RPKM)
Aicda
Tdg
Serum
2i/LIF 6h
2i/LIF 24h
2i/LIF 72h
B
WGBS
80
60
40
20
0
%5mC
0
4
8
32
Time(h)
C
TAB-Seq
5
4
3
2
1
0
%5hmC
0
4
8
32
Time(h)
D
Distribution of dyads
(measured by Hairpin-BS)
100
75
50
25
0
%CpG dyads
63%
19%
18%
31%
23%
46%
12%
7%
81%
Serum
2i 7days
2i LT
%mCpG/GpCm
%mCpG/GpC
%CpG/GpC
E
Dynamic changes in
DNMT3A/B protein levels
predicted
measured by MS (→FigS4A)
2
0
-2
-4
-6
-8
log2 (2i/serim)
0
36
72
Time(h)

# Figure S2

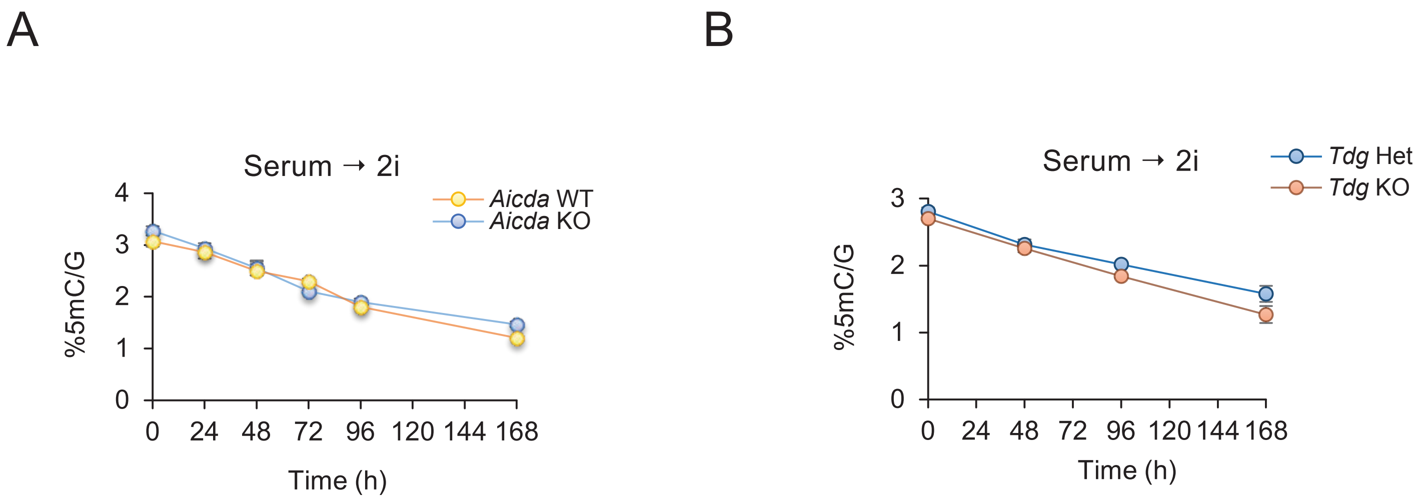
A
B
Serum → 2i
Aicda WT
Aicda KO
%5mC/G
Time (h)
0
24
48
72
96
120
144
168
Serum → 2i
Tdg Het
Tdg KO
%5mC/G
Time (h)

# Figure S3

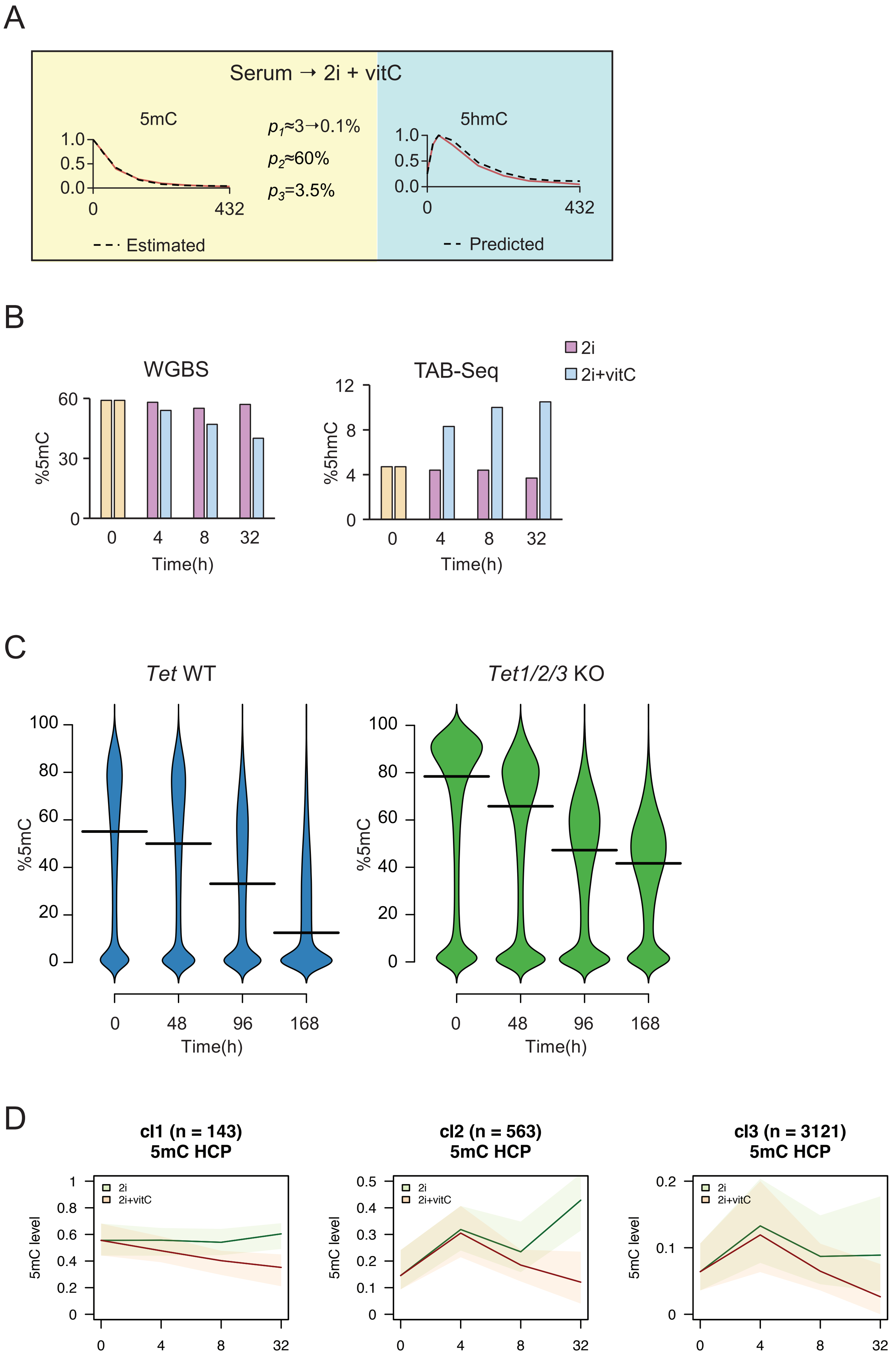
A
Serum → 2i + vitC
5mC
1.0
0.5
0.0
0
432
$p_1$≈3→0.1%
$p_2$≈60%
$p_3$=3.5%
Estimated
5hmC
1.0
0.5
0.0
0
432
Predicted
B
WGBS
%5mC
60
30
0
0
4
8
32
Time(h)
TAB-Seq
%5hmC
12
8
4
0
0
4
8
32
Time(h)
2i
2i+vitC
C
Tet WT
%5mC
100
80
60
40
20
0
0
48
96
168
Time(h)
Tet1/2/3 KO
%5mC
100
80
60
40
20
0
0
48
96
168
Time(h)
D
cl1 (n = 143)
5mC HCP
5mC level
1
0.8
0.6
0.4
0.2
0
2i
2i+vitC
0
4
8
32
cl2 (n = 563)
5mC HCP
5mC level
0.5
0.4
0.3
0.2
0.1
0
2i
2i+vitC
0
4
8
32
cl3 (n = 3121)
5mC HCP
5mC level
0.2
0.1
0
2i
2i+vitC
0
4
8
32

# Figure S4

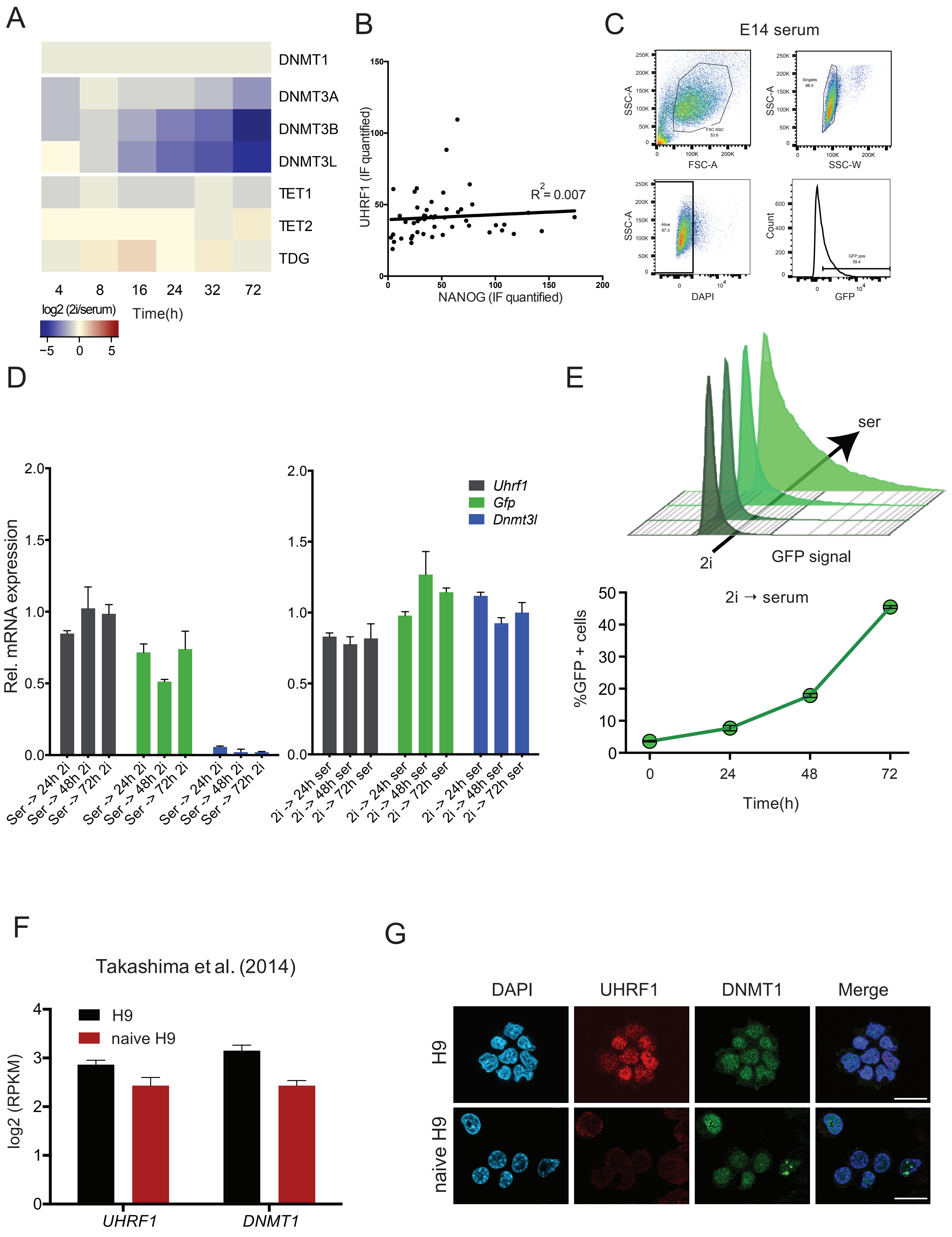

A
DNMT1
DNMT3A
DNMT3B
DNMT3L
TET1
TET2
TDG
4 8 16 24 32 72
Time(h)
log2 (2i/serum)
−5 0 5
B
UHRF1 (IF quantified)
NANOG (IF quantified)
$R^2$ = 0.007
C
E14 serum
SSC-A
FSC-A
SSC-W
DAPI
Count
GFP
D
Rel. mRNA expression
Uhrf1
Gfp
Dnmt3l
Ser -> 24h 2i
Ser -> 48h 2i
Ser -> 72h 2i
2i -> 24h ser
2i -> 48h ser
2i -> 72h ser
E
ser
2i
GFP signal
2i → serum
%GFP + cells
Time(h)
0 24 48 72
F
Takashima et al. (2014)
H9
naive H9
log2 (RPKM)
UHRF1
DNMT1
G
DAPI
UHRF1
DNMT1
Merge
H9
naive H9

# Figure S5

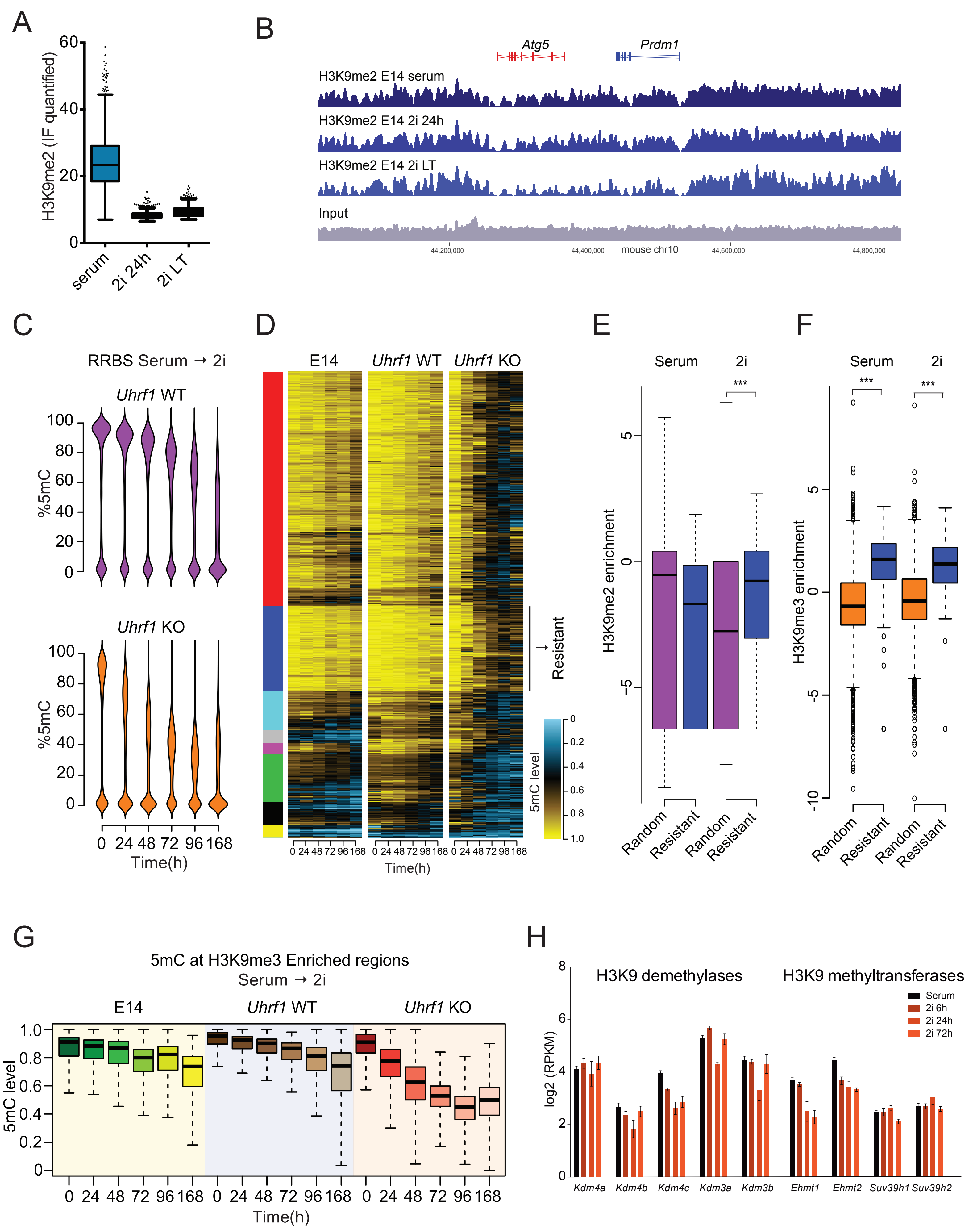

A
H3K9me2 (IF quantified)
serum
2i 24h
2i LT
B
Atg5
Prdm1
H3K9me2 E14 serum
H3K9me2 E14 2i 24h
H3K9me2 E14 2i LT
Input
44,200,000
44,400,000
mouse chr10
44,600,000
44,800,000
C
RRBS Serum → 2i
Uhrf1 WT
Uhrf1 KO
%5mC
Time(h)
D
E14
Uhrf1 WT
Uhrf1 KO
Resistant
5mC level
Time(h)
E
Serum
2i
H3K9me2 enrichment
Random
Resistant
F
Serum
2i
H3K9me3 enrichment
Random
Resistant
G
5mC at H3K9me3 Enriched regions
Serum → 2i
E14
Uhrf1 WT
Uhrf1 KO
5mC level
Time(h)
H
H3K9 demethylases
H3K9 methyltransferases
log2 (RPKM)
Serum
2i 6h
2i 24h
2i 72h
Kdm4a
Kdm4b
Kdm4c
Kdm3a
Kdm3b
Ehmt1
Ehmt2
Suv39h1
Suv39h2

# Figure S6

2nd Solution of PGC modelling
“TET-dependent demethylation”

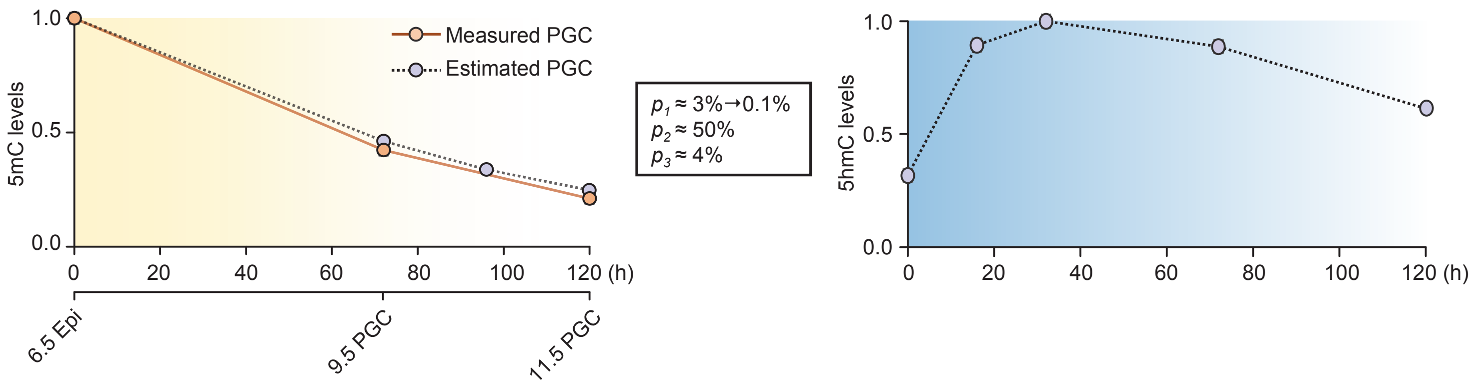

# Table S1, Related to Figure 1 and S1:

List of initial values and parameters employed in the mathematical modeling

| Input | | | |
|---|---|---|---|
| Dyads | Symbol | initial values | Value determination methods |
| CpG/GpC | $x_1$ | 177038 | Hairpin BS-seq |
| $^{m}$CpG/GpC | $x_2$ | 176187 | Hairpin BS-seq |
| $^{m}$CpG/GpC$_{m}$ | $x_3$ | 553158 | Hairpin BS-seq |
| $^{hm}$CpG/GpC$_{m}$ | $x_4$ | 55783 | Hairpin BS-seq and TAB-seq |
| $^{hm}$CpG/GpC$_{hm}$ | $x_5$ | 14991 | Hairpin BS-seq and TAB-seq |
| $^{hm}$CpG/GpC | $x_6$ | 8894 | Hairpin BS-seq and TAB-seq |
| | | | |
| Parameters | Function | | |
| $p_1$ | *De novo* methylation | | |
| $p_2$ | Maintenance methylation | | |
| $p_3$ | hydroxymethylation | | |
| $d$ | Cell division rate | | |

## SUPPLEMENTAL FIGURE LEGENDS

**Figure S1. Dynamic regulation of 5mC and 5hmC during serum-to-2i conversion of mESCs, Related to Figure 1 and Table S1**

(A) Expression level of *Aicda* and *Tdg* during the serum-to-2i transition. Error bars indicate mean ± SD from three biological replicates.
(B) Percentage of 5mC as measured by WGBS in E14 ESCs during serum-to-2i conversion.
(C) Percentage of 5hmC as measured by TAB-Seq in E14 ESCs during serum-to-2i conversion.
(D) Distribution of methylation levels in CpG dyads, measured by Hairpin-BS-Seq, in serum and 2i ESCs
(E) Dynamic changes of DNMT3A/B proteins, as predicted by the mathematical modeling (changes in $p_1$) and corresponding real protein levels, determined by protein mass spectrometry (compare Figure S4A).

**Figure S2. Global demethylation kinetics in mutants of the DNA methylation machinery, Related to Figure 2**

(A) Levels of 5mC in *Aicda* KO and controls ESCs (*Aicda* WT) during the serum-to-2i transition, measured by mass spectrometry. Error bars indicate mean ± SD from three biological replicates.
(B) Levels of 5mC in *Tdg* KO and controls ESCs (*Tdg* Het) during the serum-to-2i transition, measured by mass spectrometry. Error bars indicate mean ± SD from three biological replicates.

**Figure S3. Global demethylation kinetics in mutants of the TET proteins, Related to Figure 3 and Table S2**

(A) Mathematical modeling of 5mC and 5hmC dynamics during the serum-to-2i transition in the presence of vitC. The estimated value for active demethylation $p_3$ was estimated using measured 5mC values only. The overlay between the mathematical model prediction (dotted line) and the normalized measured (red line) 5mC and 5hmC data is shown with $p_3$=3.5%.
(B) Global changes in absolute 5mC and 5hmC levels during the first 32h of reprogramming from serum (wheat) to 2i (purple) or 2i+vitC (light blue) measured by WGBS (left panel) and TAB-seq (right panel), respectively.
(C) Bean-plot showing methylation data from RRBS-seq in *Tet* WT and *Tet1/2/3* KO datasets during the serum-to-2i transition. Horizontal bars represent the median values.
(D) 5mC levels at HCPs, subdivided into three main clusters. Shown is the overall trend and the boundaries of the *shaded* area in each plot indicate the 25th percentiles and the 75th percentiles of each cluster in E14 ESCs with and without vitC.

**Figure S4. Uhrf1 is regulated at the protein level also in human ESCs, Related to Figure 4**

(A) Relative protein levels of DNMTs and TETs determined by protein mass spectrometry signal (label-free quantification (LFQ)) at different time points during serum-to-2i conversion.
(B) Correlation of the quantified signals of UHRF1 and NANOG in immunofluorescence staining of serum ESCs. The black line represents the linear regression between UHRF1 and NANOG signals in serum ESCs.
(C) FACS gating strategy used to quantify GFP positive cells in the UHRF1-GFP fusion protein ESC line. Shown is one representative sample grown in primed serum ESC conditions.
(D) mRNA levels of *Uhrf1*, *Gfp* and *Dnmt3l* from UHRF1-GFP fusion protein ESC line during the serum-to-2i transition and the 2i-to-serum transition. Error bars indicate mean ± SD from three biological replicates.
(E) FACS analysis of UHRF1-GFP fusion protein in ESCs during 2i-to-serum conversion. Histograms show the GFP signal intensity at different time points during the conversion. The black arrow depicts the threshold used to quantify the percentage of GFP+ cells, shown in the line graph. Error bars indicate mean ± SD from three biological replicates.
(F) Graph shows the quantification of GFP+ cells at each time-point. Error bars indicate mean ± SD from three biological replicates.
(G) Expression levels of *UHRF1* and *DNMT1* in conventional and naive human ESCs. RPKM values from (Takashima et al., 2014). Error bars indicate mean ± SD.
(H) Immunofluorescence staining of DAPI (blue), UHRF1 (red) and DNMT1 (green) in conventional and naive human ESCs. Scale bar represents 20μm.

**Figure S5. UHRF1 and H3K9me2 are necessary to maintain methylation at specific loci, Related to Figure 5**

(A) Semi-quantification of H3K9me2 immunofluorescence staining in serum, 24h 2i and LT 2i ESCs. The y-axis corresponds to arbitrary units of mean values of pixel intensity.
(B) ChIP-Seq track for H3K9me2 at the *Prdm1* locus (50 kb) from serum, 24h 2i and LT 2i ESCs.
(C) Bean-plot showing methylation data from RRBS-Seq in *Uhrf1* WT and *Uhrf1* KO datasets during the serum-to-2i transition.
(D) Heatmap showing methylation levels of the genomic features that display highest degree of resistance to demethylation in *Uhrf1* KO ESCs. Blue cluster highlights a group of regions resistant to demethylation in cells with UHRF1.
(E) Enrichment of H3K9me2 and (F) H3K9me3 over regions identified to resist DNA demethylation during the serum-to-2i transition (blue cluster from (D)).
(G) Levels of 5mC, determined by RRBS at H3K9me3 enriched regions (from (Marks et al., 2012)) during the serum-to-2i transition in E14, *Uhrf1* WT and *Uhrf1* KO ESCs.
(H) Expression levels of known H3K9 modifiers. Error bars indicate mean ± SD from three biological replicates.

Student's t test was performed for the indicated comparisons. ***p<0.001

**Figure S6. Mathematical modeling of PGCs, Related to Figure 6**

Summary of the 2nd solution of the mathematical modeling of migratory PGC demethylation. The first column shows measured (solid lines) and estimated (dotted lines) global levels of 5mC (values have been scaled) in migratory PGCs. The second column summarizes the estimated activities of the three pathways. The third column shows the predicted (dotted lines) global 5hmC values (scaled).

## SUPPLEMENTAL TABLE LEGENDS

**Table S1, Related to Figure 1 and S1:** List of initial values and parameters employed in the mathematical modeling.

**Table S2, Related to Figure 3 and S3:** List of 500bp regions higher methylated in *Tet1/2/3* KO cells compared to *Tet* WT cells. Reads were mapped to the mouse NCBIM37 genome build using Bismark and average CpG methylations were calculated over each 500bp region in Seqmonk.

## SUPPLEMENTAL EXPERIMENTAL PROCEDURES

### Cell Culture

All mouse ESC lines including E14 (129/Ola), J1 (129S4/SvJae), *Tdg*$^{-/-}$ (129/C57BL6) (Cortázar et al., 2011), *Aicda*$^{-/-}$ (C57BL6), *Tet[1*$^{-/-}$*,2*$^{-/-}$*,3*$^{-/-}$*]* (C57BL6/129/FVB) (Hu et al., 2014), and their control lines were cultured without feeders in the presence of LIF in DMEM containing 15% fetal calf serum. *Dnmt1*$^{fl/fl}$ (C57BL6), *Uhrf1*$^{fl/fl}$ (C57BL6) (Sharif et al., 2016), *Dnmt[3a*$^{fl/fl}$*, 3b*$^{fl/fl}$*]* (129/Ola/C57BL6), *Tet[1*$^{-/-}$*,2*$^{-/-}$*]* (129/C57BL6) (Dawlaty et al., 2013), and their control lines were expanded on feeder and purified from feeders by sequential pre-plating. Tamoxifen inducible Cre recombinase was used to induce recombination of loxP sites in the floxed cell lines. Tamoxifen (Sigma) was added directly to the media 48 hours prior to the experiment. For serum-to-2i transition, serum medium was replaced by serum-free N2B27 (N2 & B27; Gibco) or NDiff227 (StemCells, Inc.) supplemented with LIF, MEK inhibitor PD0325901 (1 μM) and GSK3 inhibitor CHIR99021 (3 μM), together known as 2i (Ying et al., 2008). For serum-to-2i switching in the presence of vitamin C (L-ascorbic acid, Sigma), 17 μM of vitamin C (dissolved in water) was added to 2i. The media were refreshed every day and cells were passaged every 2-3 days. Primed human ESCs H9 (WiCell Research Institute) (Thomson et al., 1998) were maintained as previously described (E8 media, StemCells, Inc.) on vitronectin; naive human ESCs (H9) were obtained from Austin Smith and maintained in defined naive growth conditions (Takashima et al., 2014) on feeders.

### Generation of *Dnmt[3a*$^{fl/fl}$*, 3b*$^{fl/fl}$*]* lines

*Dnmt[3a*$^{fl/fl}$*, 3b*$^{fl/fl}$*]* cell lines were derived as previously reported (Nesterova et al., 2008) from XY embryos homozygous for conditional mutations of *Dnmt3a* and *Dnmt3b* genes (Dodge et al., 2005; Kaneda et al., 2004) and heterozygous for tamoxifen-inducible Cre-recombinase targeted into the Rosa26 locus (Vooijs et al., 2001). Eight-cell embryos were flushed from oviducts and incubated in M16 medium (Sigma-Aldrich) supplemented with PDO325901 (1μM, Miltenyi Biotec Ltd) and CHIR99021 (3μM, Miltenyi Biotec Ltd) for 24 - 48 hours. After further 24 hours of incubation in NDiff227 medium (StemCells, Inc.) supplemented with 2i and LIF, hatched blastocysts were moved individually into wells of 4-well plates and allowed to grow for 4 to 7 days until ESC-like outgrowths could be seen. Dissected and trypsinised outgrowths were seeded on 4-well plates and cell lines were expanded and genotyped by PCR.

### WGBS, RRBS, TAB-seq and Hairpin-Bisulfite Sequencing

DNA was isolated using the Cell Culture DNA Midi Kit from QIAGEN (Cat No 13343), GenElute™ Mammalian Genomic DNA Miniprep Kit (Cat No G1N350, Sigma), or Beckman Coulter's Agencourt RNAdvance Cell v2 kit. WGBS, RRBS and TAB-Seq was performed as described previously (Lister et al., 2008; Smallwood et al., 2011; Yu et al., 2012). We performed paired-end DNA sequencing (2*100 nucleotides) using the Illumina Hi-Seq 2000 for WGBS, and single-end DNA sequencing (50 nucleotides) using the Illumina Hi-Seq 2000 for RRBS. Whole genome hairpin bisulfite sequencing was performed as described (Zhao et al., 2014) and sequencing was performed on Illumina MiSeq.

### ChIP-Seq and ChIP-BS-Seq

For chromatin immunoprecipitation, cells were cross-linked with 1% methanol-free formaldehyde in fresh medium for 10 minutes. Cross-linking reaction was quenched with glycine at a final concentration of 0.2M. Cells were washed twice with ice-cold PBS and harvested. Cell pellets were resuspended in LB1 buffer (50 mM Hepes-KOH, pH 7.5; 140 mM NaCl; 1 mM EDTA;10% Glycerol; 0.5% NP-40 or Igepal CA-630; 0.25% Triton X-100, protease inhibitors) for 10 minutes at 4°C, pelleted and resuspended in LB2 buffer (10 mM Tris-HCl, pH8.0; 200 mM NaCl; 1 mM EDTA; 0.5 mM EGTA, protease inhibitors) for 10 minutes at 4°C. Cells were pelleted, resuspended in LB3 buffer (10 mM Tris-HCl, pH 8; 100 mM NaCl; 1 mM EDTA; 0.5 mM EGTA; 0.1% Na-Deoxycholate; 0.5% N-lauroylsarcosine, protease inhibitors) and sonicated using Misonix Sonicator 3000. After addition of Triton-X to a final concentration of 1%, the lysate was centrifuged at 20,000g for 10 minutes to pellet debris. Sonicated chromatin was added to the bead-antibody complexes and incubated overnight at 4°C. Bead-antibody were prepared as follows: Protein G-coupled Dynabeads were incubated overnight with primary antibodies (anti-H3K9me2; Abcam ab1220) in PBS added with 5 mg/ml BSA. Beads were washed extensively with RIPA buffer (50mM HEPES pH 7.6, 1mM EDTA, 0.7% Na-Deoxycholate, 1% NP-40, 0.5M LiCl), once with 1x TE buffer and eluted in 200ul of buffer containing 1% SDS and 0.1M NaHCO3. Cross-linking was reversed by incubation at 65°C overnight, followed by RNAse A treatment at 37°C for 1 hour and Proteinase K treatment at 55°C for 2 hours. DNA was then extracted using Beckman Coulter's Agencourt RNAdvance Cell v2 kit following the manufacture's

instructions. Eluted DNA was then used for ChIP-Seq library preparation using the MicroPlex Library Preparation Kit v2 (Diagenode) or alternatively treated as WGBS samples as described above.

**Proteomics**
Total proteins for each sample were isolated and tryptic digested following a published label-free proteomics protocol (Liu et al., 2012). Protein identification and quantification were performed using MaxQuant software (version 1.3.5.7) with standard settings (Cox and Mann, 2008) and search against the UniProtKB/Swiss-Prot human database (generated from version 06-2012).

**RNA-Seq and Reverse Transcription Quantitative Real-Time PCR**
Total RNA for each sample was extracted using Beckman Coulter's Agencourt RNAdvance Cell v2 kit following the manufacture's instructions. Extracted RNA was PolyA-enriched and used for library preparation using the TruSeq RNA Library Prep Kit v2 (Illumina) following the manufacturer's instructions. Libraries were indexed using Illumina Indexes and 50 bp single-end sequencing was performed on Illumina HiSeq 2000 instruments using TruSeq reagents (Illumina, San Diego, CA, USA), according to manufacturer's instructions. Quantitative PCR experiments were performed following standard protocols. Complementary DNA (cDNA) was prepared from DNAse treated total RNA using SuperScript III (Invitrogen) and oligo-dT primers. At least two independent samples were assayed for all quantitative PCR reactions. Endogenous controls (*Atp5b*, *Hspcb*) were used to normalize expression. Primers are listed below.

| Gene | Primer | Sequence |
|---|---|---|
| *Uhrf1* | msRT_UHRF1_F | GCTCCAGTGCCGTTAAGACC |
| | msRT_UHRF1_R | CACGAGCACGGACATTCTTG |
| *Gfp* | RT_eGFP_F | CAACAGCCACAACGTCTATATCAT |
| | RT_eGFP_R | ATGTTGTGGCGGATCTTGAAG |
| *Hspcb* | msRT_Hspcb_F | GCTGGCTGAGGACAAGGAGA |
| | msRT_Hspcb_R | CGTCGGTTAGTGGAATCTTCATG |
| *Atp5b* | msRT_Atp5b_F | GGCCAAGATGTCCTGCTGTT |
| | msRT_Atp5b_R | GCTGGTAGCCTACAGCAGAAGG |
| *Dnmt3l* | msRT_DNMT3L_F | ATGGACAATCTGCTGCTGACTG |
| | msRT_DNMT3L_R | CGCATAGCATTCTGGTAGTCTCTG |

**Analysis of nucleoside mass spectrometry**
Nucleosides levels were quantified by integrating peak areas for the fragment ions from extracted mass spectrometry ion chromatograms of the relevant scans and calibrated relative to external standards obtained by digestion of nucleotide triphosphates.

**Fluorescence-activated cell sorting (FACS)**
A stable mouse ESC line, overexpressing UHRF1 fused to GFP, was generated. Stable integration of the exogenous DNA was selected for with blasticidin, which is expressed from the same promoter as *Uhrf1-Gfp* using an internal ribosome entry site (IRES) connecting the two cDNAs. UHRF1-GFP cells were grown under serum or 2i conditions. Media was switched to 2i or serum, respectively, and cells were cultured for the indicated time periods. Cells were then dissociated into single cells and analyzed on a LSR Fortessa Cell Analyzer (BD). Cells were gated for singlets and living cells were identified using the level of DAPI incorporation and the level of GFP signal was recorded for each cell. The expression of GFP in long-term 2i conditions was set as negative background and the percentage of cells with higher GFP signal in each condition was plotted.

**Immunofluorescence and Imaging**
Antibody staining was performed as previously described (Santos et al., 2003) on ESCs grown on coverslips or cytospun, after fixation with 2% PFA for 30 minutes at room temperature. Briefly, cells were permeabilised with 0.5% TritonX-100 in PBS for 1h; blocked with 1% BSA in 0.05%Tween20 in PBS (BS) for 1h; incubation of the appropriate primary antibody diluted in BS; followed by wash in BS and secondary antibody. For simultaneous detection with DNA methylation, after first round of antibody staining, samples were washed in PBS, post-fixed in 2% PFA for 10 minutes, treated with 2N HCl for 30 minutes at 37ºC and washed extensively with PBS before incubating with anti-5mC, diluted in BS. All secondary antibodies were Alexa Fluor conjugated (Molecular Probes) diluted 1:1000 in BS and incubated for 30 minutes. Incubations were performed at room temperature unless otherwise

stated. Staining of replication foci was performed by adding 10µM EdU for 8 minutes to the culture medium prior to fixation and visualized with a Click-iT EdU Imaging kit (Molecular Probes). DNA was counterstained with 5µg/mL DAPI in PBS. Single optical sections were captured with a Zeiss LSM780 microscope (63x oil-immersion objective) and the images pseudo-colored using Adobe Photoshop. For visualization, images were corrected for brightness and contrast, within the recommendations for scientific data. Single cell RGB profiles were plotted with ImageJ 1.44p (NIH) after removing the background around the cells. Fluorescence semi-quantification analyses were performed with Volocity 6.3 (Improvision). Antibodies used are listed below.

| Antibody | Cat.no. | Company | Dilution |
|---|---|---|---|
| Anti-mUHRF1 (Th-10a) | D289-3 | MBL | 1:250 |
| Anti-hUHRF1 (H-8) | sc-373750 | Santa Cruz | 1:100 |
| Anti-DNMT1 | ab87654 | Abcam | 1:1000 |
| Anti-H3K9me2 | ab1220 | Abcam | 1:200 |
| Anti-5mC | BI-MECY-0100 | Eurogentec | 1:500 |
| Anti-NANOG | ab80892 | Abcam | 1:500 |

**Western blot analysis**
Whole cell protein extracts were prepared using 1xRIPA buffer (Thermo Scientific, 89900) with protease and phosphatase inhibitors (Fisher Scientific, PN87786 and PN78420). 10 µg of proteins were resolved by SDS-PAGE and transferred on nitrocellulose membranes. Membranes were blocked in PBS-0.1%Tween (PBST) containing 5% BSA (blocking buffer) overnight at 4˚C. Primary antibody incubation was done at room temperature for 2 hours, followed by two washes with PBST and incubated with HRP conjugated secondary antibodies (1:6000) in blocking buffer. HRP conjugates were detected with enhanced chemiluminescence (ECL, Amersham Biosciences). Antibodies used are listed below.

| Antibody | Cat.no. | Company | Dilution |
|---|---|---|---|
| Anti-mUHRF1 (Th-10a) | sc-98817 | Santa Cruz | 1:1000 |
| Anti-H3K9me2 | 07-212 | Millipore | 1:1000 |
| Anti-HSP90 (H-114) | sc-7947 | Santa Cruz | 1:1000 |

**Bioinformatics**
Raw sequence reads were trimmed to remove poor quality reads, adapter contamination, and the first 6 base pairs, using Trim Galore (http://www.bioinformatics.babraham.ac.uk/projects/trim_galore/).
For RRBS, WGBS, TAB-Seq, and ChIP-BS-Seq, the remaining sequences were mapped to the mouse reference genome NCBIM37 using Bismark (Krueger and Andrews, 2011) or rmapbs-pe (Song et al., 2013). CpG methylation calls were extracted and analyzed using SeqMonk (www.bioinformatics.babraham.ac.uk/projects/seqmonk/) and/or custom R scripts. For TAB-Seq, hmC protection/non-protection and mC non-conversion rates were quantified and estimated levels were corrected as described in detail previously (Lister et al., 2013; Yu et al., 2012). A maximum likelihood method (Qu et al., 2013) on the combined data from WGBS and TAB-Seq were used to estimate absolute levels for 5mC and 5hmC.
For RNA-Seq data analysis, trimmed sequencing reads were aligned to mouse genome assembly GRCm38 using TopHat. SeqMonk, and custom R scripts were used to normalize the data and perform pair-wise comparisons of genes at each time point.
For ChIP-Seq data analysis, trimmed sequencing reads were aligned to mouse genome assembly NCBIM37 using Bowtie2 and analyzed using SeqMonk and/or custom R scripts.

**Mathematical modeling**
The structure (equations) of the model was adapted from (McGovern et al., 2012). Equations were solved numerically using the Runge-Kutta 4$^{th}$ order method. We implemented the code in Perl and parallelized using the Many-Core Engine (MCE) Perl module (https://code.google.com/p/many-core-engine-perl/).
As input values, the percentages of unmethylated CpG dyads (CpG/GpC; $x_1$), hemi-methylated CpG dyads (mCpG/GpC; $x_2$) and fully methylated CpG dyads (mCpG/GpCm; $x_3$) were extracted directly from WGBS and hairpin BS-Seq. Total percentage of hmCpG was directly calculated from TAB-seq data. Due to the lack of hairpin TAB-seq data, we estimated hemi-hydroxymethylated CpG (hmCpG/GpC; $x_4$), hemi-methylated hemi-hydroxymethylated CpG dyads (mCpG/GpChm; $x_5$) and

fully hydroxymethylated CpG (hmCpG/GpChm, x6), by considering the fraction of methylated/hemimethylated CpG compared to total mCpG dyads and the proportion of total mCpG to the total hmCpG dyads.

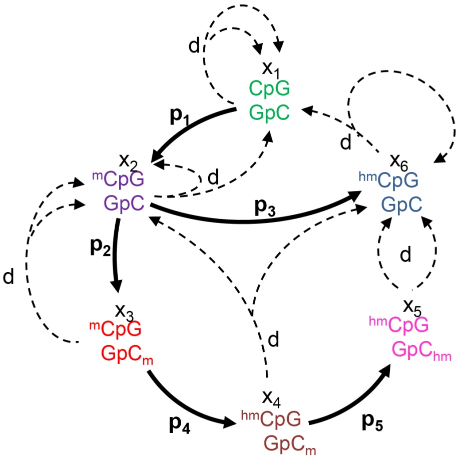


$$\frac{\partial x_1}{\partial t} = (d - l - p_1)x_1 + dx_2 + dx_6$$
$$\frac{\partial x_2}{\partial t} = p_1x_1 - (l + p_2 + p_3)x_2 + 2dx_3 + dx_4$$
$$\frac{\partial x_3}{\partial t} = p_2x_2 - (d + l + p_4)x_3$$
$$\frac{\partial x_4}{\partial t} = p_4x_3 - (d + l + p_5)x_4$$
$$\frac{\partial x_5}{\partial t} = p_5x_4 - (d + l)x_5$$
$$\frac{\partial x_6}{\partial t} = p_3x_2 + dx_4 + 2dx_5 - lx_6$$

Four types of parameters have been defined in the model:

$\boldsymbol{p_1}$: proportionality constant for *de novo* methylation
$\boldsymbol{p_2}$: proportionality constant for maintenance methylation
$\boldsymbol{p_{3\text{-}5}}$: proportionality constants for hydroxymethylation ($p_3$=$p_4$/2=$p_5$)
**Although in theory, $p_4$ seems to be twice of $p_3$ or $p_5$, our simulations with different combinations of parameters shows that for the best fits these values adopt almost similar values. Therefore, during our simulations we assumed $p_3$=$p_4$=$p_5$.
***d***: cell division rate (population growth rate) is the number of doublings that occur per unit of time and is calculated as follow:

$$T_d = \frac{\log(2)}{\log(1+d)} \approx \frac{0.7}{d} \Rightarrow d \approx 0.7 / T_d \quad \mathrm{T_d} = \text{doubling time}$$

For serum and 2i, the doubling time was set to 13 and 16, respectively. Adaptation of cell cycle (doubling time) during serum to 2i transition occurs quite fast (after ~8h; unpublished data), therefore we set doubling time/growth rate before and after 8h as serum and 2i, respectively.
To estimate the parameters, a hypothetical parameter space ($\theta_p$) was defined encompassing every combination of the following setting: $\theta_{p_1}$ and $\theta_p$ oscillating in the range of $0 \leq \theta_{p_{1,3-5}} \leq 1$ with step-size 0.00125. To reduce the dimensionality, we restricted $\theta_{p_2}$ to vary in the range of $0 \leq \theta_{p_2} \leq 1$ with step-size of 0.05. We implemented dynamic changing instead of single, non-variable $p_1$-$p_5$ constants to mimic the anticipated gradual change in protein abundance and/or enzymatic activity throughout the interconversion. We divided the time-course into non-overlapping time segments from 0-4h, 4-8h, 8-16h, 16-24h, 24-32h, 32-72h, 72-144h, 144-432h. Consequently, whenever a number from the parameter space is assigned to the initial $p_1$-$p_5$ values, these values are dynamically changed in each subsequent time interval to reflect the predicted fold change in protein abundances.
The simulations were performed with all combinations of the parameter spaces ($\theta_p$ and $f_k$) and for each set of parameters, the percentages of dyads (the level of 5mC as well as 5hmC) was generated for every point throughout the time course. Then, using the least square optimization we compared measured data to all simulated data.
The least squares method finds its optimum when the sum, S, of squared residuals

$$S = \sum_{i=1}^{n} (measured_i - predicted_i)^2$$

is a minimum. A residual is defined as the difference between the actual value of the dependent variable and the value predicted by the model.
Simulated profiles with the least minimum error values were selected and the median of their related parameters were considered as the optimal parameters for further predictions.

## References


Cortázar, D., Kunz, C., Selfridge, J., Lettieri, T., Saito, Y., MacDougall, E., Wirz, A., Schuermann, D., Jacobs, A.L., Siegrist, F., et al. (2011). Embryonic lethal phenotype reveals a function of TDG in maintaining epigenetic stability. Nature *470*, 419–423.

Cox, J., and Mann, M. (2008). MaxQuant enables high peptide identification rates, individualized p.p.b.-range mass accuracies and proteome-wide protein quantification. Nat Biotechnol *26*, 1367–1372.

Dawlaty, M.M., Breiling, A., Le, T., Raddatz, G., Barrasa, M.I., Cheng, A.W., Gao, Q., Powell, B.E., Li, Z., Xu, M., et al. (2013). Combined Deficiency of Tet1 and Tet2 Causes Epigenetic Abnormalities but Is Compatible with Postnatal Development. Dev Cell *24*, 310–323.

Dodge, J.E., Okano, M., Dick, F., Tsujimoto, N., Chen, T., Wang, S., Ueda, Y., Dyson, N., and Li, E. (2005). Inactivation of Dnmt3b in mouse embryonic fibroblasts results in DNA hypomethylation, chromosomal instability, and spontaneous immortalization. J Biol Chem *280*, 17986–17991.

Hu, X., Zhang, L., Mao, S.-Q., Li, Z., Chen, J., Zhang, R.-R., Wu, H.-P., Gao, J., Guo, F., Liu, W., et al. (2014). Tet and TDG mediate DNA demethylation essential for mesenchymal-to-epithelial transition in somatic cell reprogramming. Cell Stem Cell *14*, 512–522.

Kaneda, M., Okano, M., Hata, K., Sado, T., Tsujimoto, N., Li, E., and Sasaki, H. (2004). Essential role for de novo DNA methyltransferase Dnmt3a in paternal and maternal imprinting. Nature *429*, 900–903.

Krueger, F., and Andrews, S.R. (2011). Bismark: a flexible aligner and methylation caller for Bisulfite-Seq applications. Bioinformatics *27*, 1571–1572.

Lister, R., Mukamel, E.A., Nery, J.R., Urich, M., Puddifoot, C.A., Johnson, N.D., Lucero, J., Huang, Y., Dwork, A.J., Schultz, M.D., et al. (2013). Global epigenomic reconfiguration during mammalian brain development. Science *341*, 1237905–1237905.

Lister, R., O'Malley, R.C., Tonti-Filippini, J., Gregory, B.D., Berry, C.C., Millar, A.H., and Ecker, J.R. (2008). Highly integrated single-base resolution maps of the epigenome in Arabidopsis. Cell *133*, 523–536.

Liu, N.Q., Braakman, R.B.H., Stingl, C., Luider, T.M., Martens, J.W.M., Foekens, J.A., and Umar, A. (2012). Proteomics pipeline for biomarker discovery of laser capture microdissected breast cancer tissue. J Mammary Gland Biol Neoplasia *17*, 155–164.

Marks, H., Kalkan, T., Menafra, R., Denissov, S., Jones, K., Hofemeister, H., Nichols, J., Kranz, A., Stewart, A.F., Smith, A., et al. (2012). The transcriptional and epigenomic foundations of ground state pluripotency. Cell *149*, 590–604.

McGovern, A.P., Powell, B.E., and Chevassut, T.J.T. (2012). A dynamic multi-compartmental model of DNA methylation with demonstrable predictive value in hematological malignancies. J. Theor. Biol. *310*, 14–20.

Nesterova, T.B., Popova, B.C., Cobb, B.S., Norton, S., Senner, C.E., Tang, Y.A., Spruce, T., Rodriguez, T.A., Sado, T., Merkenschlager, M., et al. (2008). Dicer regulates Xist promoter methylation in ES cells indirectly through transcriptional control of Dnmt3a. Epigenetics Chromatin *1*, 2.

Qu, J., Zhou, M., Song, Q., Hong, E.E., and Smith, A.D. (2013). MLML: consistent simultaneous estimates of DNA methylation and hydroxymethylation. Bioinformatics *29*, 2645–2646.

Santos, F., Zakhartchenko, V., Stojkovic, M., Peters, A., Jenuwein, T., Wolf, E., Reik, W., and Dean, W. (2003). Epigenetic marking correlates with developmental potential in cloned bovine preimplantation embryos. Curr Biol *13*, 1116–1121.

Sharif, J., Endo, T.A., Nakayama, M., Karimi, M.M., Shimada, M., Katsuyama, K., Goyal, P., Brind'Amour, J., Sun, M.-A., Sun, Z., et al. (2016). Activation of Endogenous Retroviruses in Dnmt1−/− ESCs Involves Disruption of SETDB1-Mediated Repression by NP95 Binding to Hemimethylated DNA. Cell Stem Cell.

Smallwood, S.A., Tomizawa, S.-I., Krueger, F., Ruf, N., Carli, N., Segonds-Pichon, A., Sato, S., Hata, K., Andrews, S.R., and Kelsey, G. (2011). Dynamic CpG island methylation landscape in oocytes and preimplantation embryos. Nat Genet *43*, 811–814.

Song, Q., Decato, B., Hong, E.E., Zhou, M., Fang, F., Qu, J., Garvin, T., Kessler, M., Zhou, J., and Smith, A.D. (2013). A reference methylome database and analysis pipeline to facilitate integrative and comparative epigenomics. PLoS ONE *8*, e81148.

Takashima, Y., Guo, G., Loos, R., Nichols, J., Ficz, G., Krueger, F., Oxley, D., Santos, F., Clarke, J., Mansfield, W., et al. (2014). Resetting Transcription Factor Control Circuitry toward Ground-State

Pluripotency in Human. Cell *158*, 1254–1269.

Thomson, J.A., Itskovitz-Eldor, J., Shapiro, S.S., Waknitz, M.A., Swiergiel, J.J., Marshall, V.S., and Jones, J.M. (1998). Embryonic stem cell lines derived from human blastocysts. Science *282*, 1145–1147.

Vooijs, M., Jonkers, J., and Berns, A. (2001). A highly efficient ligand-regulated Cre recombinase mouse line shows that LoxP recombination is position dependent. EMBO Rep *2*, 292–297.

Ying, Q.-L., Wray, J., Nichols, J., Batlle-Morera, L., Doble, B., Woodgett, J., Cohen, P., and Smith, A. (2008). The ground state of embryonic stem cell self-renewal. Nature *453*, 519–523.

Yu, M., Hon, G.C., Szulwach, K.E., Song, C.-X., Zhang, L., Kim, A., Li, X., Dai, Q., Shen, Y., Park, B., et al. (2012). Base-resolution analysis of 5-hydroxymethylcytosine in the mammalian genome. Cell *149*, 1368–1380.

Zhao, L., Sun, M.-A., Li, Z., Bai, X., Yu, M., Wang, M., Liang, L., Shao, X., Arnovitz, S., Wang, Q., et al. (2014). The dynamics of DNA methylation fidelity during mouse embryonic stem cell self-renewal and differentiation. Genome Research *24*, 1296–1307.
